# Environmental pollution and social factors as contributors to preterm birth in Fresno County

**DOI:** 10.1186/s12940-018-0414-x

**Published:** 2018-08-29

**Authors:** Amy M. Padula, Hongtai Huang, Rebecca J. Baer, Laura M. August, Marta M. Jankowska, Laura L. Jellife-Pawlowski, Marina Sirota, Tracey J. Woodruff

**Affiliations:** 1 Department of Obstetrics, Gynecology and Reproductive Sciences, University of California, 550 16th Street, Mail Stop 0132, San Francisco, CA 94143 USA; 2 Department of Pediatrics, University of California, San Francisco, USA; 3 Department of Pediatrics, University of California, San Diego, USA; 4 Office of Environmental Health Hazard Assessment, California Environmental Protection Agency, Sacramento, USA; 5 Calit2/Qualcomm Institute, University of California, San Diego, USA; 6 Department of Epidemiology and Biostatistics, University of California, San Francisco, USA

**Keywords:** Preterm birth, Environmental exposure, Social factors, Prematurity, Pollution

## Abstract

**Background:**

Environmental pollution exposure during pregnancy has been identified as a risk factor for preterm birth. Most studies have evaluated exposures individually and in limited study populations.

**Methods:**

We examined the associations between several environmental exposures, both individually and cumulatively, and risk of preterm birth in Fresno County, California. We also evaluated early (< 34 weeks) and spontaneous preterm birth. We used the Communities Environmental Health Screening Tool and linked hospital discharge records by census tract from 2009 to 2012. The environmental factors included air pollution, drinking water contaminants, pesticides, hazardous waste, traffic exposure and others. Social factors, including area-level socioeconomic status (SES) and race/ethnicity were also evaluated as potential modifiers of the relationship between pollution and preterm birth.

**Results:**

In our study of 53,843 births, risk of preterm birth was associated with higher exposure to cumulative pollution scores and drinking water contaminants. Risk of preterm birth was twice as likely for those exposed to high versus low levels of pollution. An exposure-response relationship was observed across the quintiles of the pollution burden score. The associations were stronger among early preterm births in areas of low SES.

**Conclusions:**

In Fresno County, we found multiple pollution exposures associated with increased risk for preterm birth, with higher associations among the most disadvantaged. This supports other evidence finding environmental exposures are important risk factors for preterm birth, and furthermore the burden is higher in areas of low SES. This data supports efforts to reduce the environmental burden on pregnant women.

**Electronic supplementary material:**

The online version of this article (10.1186/s12940-018-0414-x) contains supplementary material, which is available to authorized users.

## Background

Preterm birth (before 37 weeks gestation) is estimated to impact 10% of U.S. births annually with resultant potential for developmental and long-term adverse health consequences [1–4]. The estimated overall cost of preterm birth in the U.S. is approximately $26.2 billion per year [5]. Preterm birth is a complex phenotype with no single known mechanism or therapeutic strategy. Causes of preterm birth have remained largely unknown [5] and therefore, in most instances, not amenable to effective interventions or prevention.

Several studies have identified important environmental risk factors for preterm birth including prenatal exposure to air pollution [6, 7], contaminated water [8–12], pesticides [13–15], traffic density (i.e. counts of motor vehicles within a given radius) [16], air toxins [17], and persistent organic pollutants [18]. In general, these studies have been relatively small and usually contaminants have been examined in isolation. Disparities in preterm birth have also been shown to exist by socioeconomic status (SES) wherein those with lower SES experience higher rates of preterm birth and other adverse pregnancy outcomes [19, 20].

Studies have also shown that exposures to pollutants differ by race/ethnicity and SES [21]. In previous work, we demonstrated that there are racial/ethnic disparities in exposure to air pollution during pregnancy [22]. Woodruff et al. found that Hispanic, African American and Asian/Pacific Islander mothers in the U.S. experienced higher mean levels of air pollution and were more than twice as likely to live in the most polluted counties compared with non-Hispanic white mothers after controlling for maternal risk factors, region and educational status [22]. Pregnant women who are exposed to multiple environmental chemicals and multiple psychosocial stressors such as neighborhood SES are at greater risk of adverse birth outcomes [23, 24]. The cumulative impacts and potential interactions between elevated exposures to chemical and psychosocial stressors have been referred to as a form of “double jeopardy” [25]. In other words, not only are such women at increased risk due to more cumulative risk factors, but the combination of risk factors is compounding the risk in a multiplicative rather than additive way. In a previous study, we found interactive effects of air pollution and SES that contribute to risk of preterm birth in the San Joaquin Valley of California [26].

Fresno County, in the San Joaquin Valley of California (CA), is an area of known environmental pollution burden [27] and a high prevalence of preterm birth (12.1% compared to 9.6% in CA in 2012). Additionally, Fresno County is characterized by diverse race/ethnicity and SES with a majority of the population being of non-white race and of lower SES, which may impact adverse health effects in conjunction with environmental exposure. Our study examines the association between multiple environmental, medical and social factors and preterm birth in Fresno County, CA from 2009 to 2012. Few studies have addressed how these factors may compound one another to contribute to preterm birth. The interaction of environmental, medical and social stressors may be critical in elucidating disparities in preterm birth. Furthermore, uncovering such compounding effects may focus policy and intervention efforts at reducing pollution burden in the most vulnerable communities.

## Methods

### Study population

Birth outcome and maternal demographic information were collected from a linked hospital discharge birth cohort database maintained by the CA Office of Statewide Health Planning and Development (OSHPD) that includes linked information from the State of CA vital records and hospital discharge records (comorbidities were identified from codes in the form of ICD-9-CM diagnoses). From this linked dataset, the study includes race/ethnicity, infant sex, maternal age at delivery, years of education, participation in the Women, Infants, and Children (WIC) food and nutrition service (a Federally-funded supplemental program), payer for delivery costs (i.e., heath insurance status), place of mother’s birth, body mass index (BMI) calculated from maternal height and pre-pregnancy weight, preexisting diabetes (ICD-9 code 250 and 648.0), gestational diabetes (648.8), preexisting hypertension (642.0, 642.1, 642.2, 642.7), gestational hypertension (642.3), preeclampsia/eclampsia (642.4, 642.4, 642.6), infection (646.5, 646.6, 647), anemia (648.2), mental illness (648.4), reported smoking, reported drug abuse, reported alcohol dependence, trimester when prenatal care began, parity, previous preterm birth, previous cesarean section, inter-pregnancy interval, premature rupture of membranes (658.1), mode of delivery (cesarean or vaginal), birth weight, birth date and gestational age at delivery (best obstetric estimate).

The sample was restricted to live-born singleton births with known birth date, birth weight between three standard deviations of mean by week of gestation [28] and gestational age between 20 and 44 weeks with complete information including census tract or zip code and births between 2009 and 2012 in Fresno County, CA.

Methods and protocols for the study were approved by the Committee for the Protection of Human Subjects within the Health and Human Services Agency of the State of California.

#### CalEnviroScreen

We used the California Communities Environmental Health Screening Tool (CalEnviroScreen 2.0, released in 2014) to estimate environmental exposures for each census tract in Fresno County [29]. The CalEnviroScreen was developed by CA’s Environmental Protection Agency’s (CalEPA) Office of Environmental Health Hazard Assessment to evaluate the cumulative existence of multiple pollutants and stressors in communities [30]. CalEnviroScreen is used to identify communities disproportionately burdened by cumulative impacts and identify disadvantaged communities for allocation of cap and trade funds generated under the Global Warming Solutions Act of 2006 [31]. CalEnviroScreen combines multiple sets of data on pollutants and stressors within a census tract into an overall index, which can be used to screen for places with the highest cumulative burdens (https://oehha.ca.gov/calenviroscreen).

**Fig. 1:**
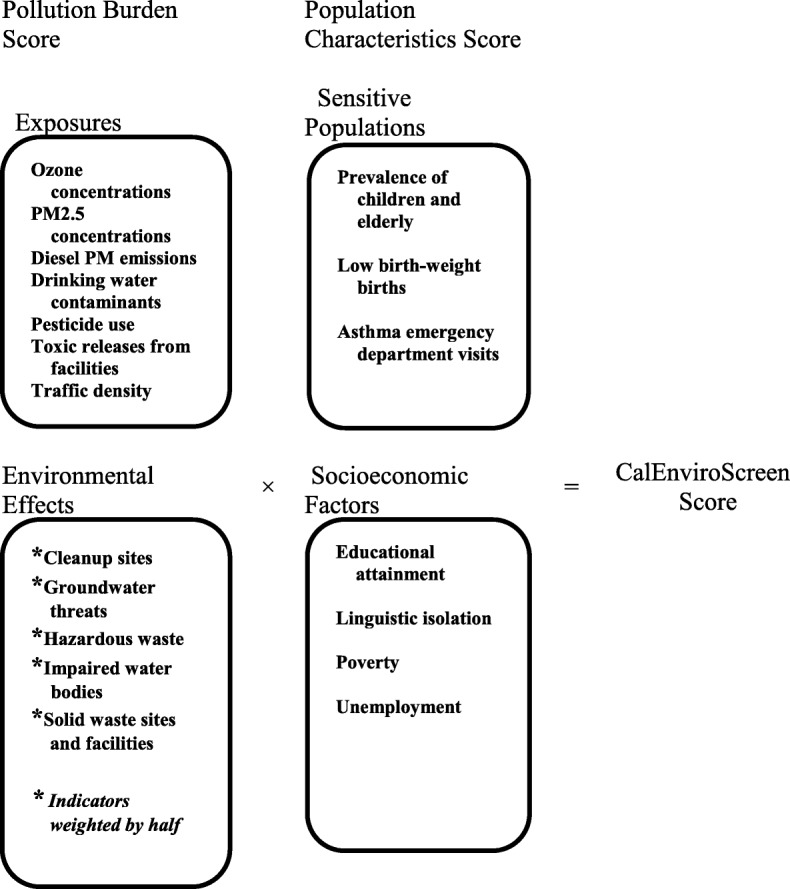
Components of the CalEnviroScreen 2.0

CalEnviroScreen 2.0 consists of 19 environmental and population indicators in total, which are aggregated into a final, relative CalEnviroScreen Score (Table 1, Fig. 1). The CalEnviroScreen Score is made up of two key categories and four components of census tract-level indicators: Pollution Burden – Exposures score and Environmental Effects; and Population Characteristics – Sensitive Populations and Socioeconomic Factors (Fig. 1). Exposures score indicators include measures of pollutant sources, releases and environmental concentrations. Environmental Effects indicators are measures of threats to the environment and degraded ecosystems caused by pollution. In calculating the average Pollution Burden, the Environmental Effects indicators are weighted by half because CalEPA considers the Exposures score indicators to be more direct measures of exposures to pollution (e.g., air pollution monitoring). These indicators likely contribute more to a person’s total pollution burden than the impact of living near contaminated land or water, where the exposure is less immediate. Indicators of Sensitive Populations and Socioeconomic Factors include both biological traits (e.g., age and health conditions of tract residents) and factors related to tract-level SES (e.g., poverty and education) that can increase susceptibility to the adverse health impacts of pollutants. These together form the Population Characteristics score. The Pollution Burden and Population Characteristics scores are then multiplied together to arrive at a final relative CalEnviroScreen score ranging from 0 to 100. The indicators are ranked into percentiles, which allows them to be compared across the state. The indicator percentiles and component scores are also useful to evaluate and understand the key drivers of vulnerability in a community. The methodology and rationale for each specific indicator is described in detail in the CalEnviroScreen 2.0 report [31]. In addition, the individual drinking water contaminants are shown in Table 1. We used the Socioeconomic Factors score from the CalEnviroScreen, which includes the following variables derived from the US Census American Community Survey: educational attainment, linguistic isolation (households where no one over 14 years of age speaks English very well), poverty and unemployment.

**Table 1:** Description of pollution indicators in CalEnviroScreen 2.0

		Indicators	Description
		Pollution Burden	Average of percentiles from *Exposure* and *Environmental Effects* indicators, with a half weighting for the Environmental Effects indicators)
Pollution Burden	Exposures	Ozone	Amount of daily maximum 8-h Ozone concentration (ppm)
		PM_2.5_	Annual mean particulate matter < 2.5 μm concentrations (μg/m^3^)
		Diesel PM	Diesel PM emissions from on-road and non-road sources (kg/day)
		Pesticides	Total pounds of selected active pesticide ingredients (filtered for hazard and volatility) used in production-agriculture per square mile in the census tract
		Toxic Release	Toxicity-weighted concentrations of modeled chemical releases to air from facility emissions and off-site incineration
		Traffic	Traffic density, in vehicle-kilometers per hour per road length, within 150 m of the census tract boundary
	Individual Drinking Water Contaminants of Violation Measures	Drinking Water Score	Drinking water contaminant index for selected contaminants
		Arsenic	Arsenic average (ppb)
		Cadmium	Cadmium average (ppb)
		DBCP	1,2-Dibromo-3-chloropropane average (ppb)
		Lead	Lead average (ppb)
		Nitrate	Nitrate (as NO_3_) average (ppm)
		Perchlorate	Perchlorate average (ppb)
		TCE	Trichloroethylene average (ppb)
		TCP	1,2,3-trichloropropane average (ppb)
		THM	Total trihalomethane average (ppb)
		Uranium	Uranium average (PCI/L)
		MCL Violations	The total number of Maximum Contaminant Level (MCL) violations for any chemical by system from 2008 to 2012 population weighted to the census tract
		TCR Violations	Total coliform rule violations by system from 2008 to 2012 population weighted to the census tract
	Environmental Effects	Groundwater Threats	Groundwater threats, sum of weighted GeoTracker leaking underground storage tank sites within buffered distances to populated blocks of census tracts
		Hazardous Waste	Sum of weighted hazardous waste facilities and large quantity generators within buffered distances to populated blocks of census tracts
		Impaired Water Bodies	Impaired water bodies, sum of number of pollutants across all impaired water bodies within buffered distances to populated blocks of census tracts
		Solid Waste	Sum of weighted solid waste sites and facilities within buffered distances to populated blocks of census tracts
		Cleanup Sites	Cleanup sites, sum of weighted EnviroStor cleanup sites within buffered distances to populated blocks of census tracts
Socioeconomic Factors		Poverty	Percent of population living below two times the federal poverty level
		Unemployment	Percent of the population over the age of 16 that is unemployed and eligible for the labor force
		Housing Burden	Percent housing burdened low income households
		Linguistic Isolation	Percent limited English speaking households

We merged the OSHPD birth records with CalEnviroScreen 2.0 data by 2010 census tract. When birth records contained 2000 census tracts, we used the relationship files for 2000 to 2010 census tracts to create area-weighted values for the CalEnviroScreen variables [32]. If a census tract identifier for a birth record was missing or invalid, zip codes were used as surrogate and similar area-weighted adjustments were made using zip code to census tract relationship files (*N* = 1879; 3.5%).

### Statistical analyses

Our primary outcome was preterm birth was defined as birth at less than 37 weeks gestation. We examined 24 exposure variables, which included the following scores and indicators from the CalEnviroScreen: Pollution Burden Score; Exposures score (component of Pollution Burden); Environmental Effects (component of Pollution Burden); 11 indicators (6 Exposures and 5 Environmental Effects); and 10 subcategories of the drinking water indicator (Fig. 1, Table 1). Each exposure variable was examined separately and classified dichotomously (split at the median) and by quintiles. We calculated Pearson correlation coefficients between the each of the indicators and scores from the CalEnviroScreen.

We examined several sets of covariates and their relationships to preterm birth and exposure indicators, which included socioeconomic variables (maternal education, payer for delivery), demographic characteristics (race/ethnicity, maternal age, maternal country of birth), obstetrical-related variables (diabetes, hypertension, smoking/alcohol/drug use during pregnancy, BMI, parity), and, among multiparous women, previous caesarean section, previous preterm birth, and inter-pregnancy interval from the previous live birth to the estimated conception of the index pregnancy. Inter-pregnancy interval was calculated from previous live birth (month and year) as reported in linked records and estimated as months to conception of the index pregnancy. Given that the day of previous live birth was not available, the middle of the month was used for calculation purposes [33]. We explored the association between the covariates and both the outcome (preterm birth) and exposure (above median levels of Pollution Burden).

We used logistic regression to evaluate the association between each indicator and preterm birth (< 37 weeks) and early preterm birth (< 34 weeks), comparing each of the higher 4 quintiles to the lowest to allow for non-monotonic relationships across the pollution distribution. We ran three sets of models: crude, adjusted with a priori variables and a stepwise selection. The covariates determined a priori included maternal education, age, race/ethnicity, and payer of delivery costs. The stepwise procedure included a forward and backward algorithm to estimate the association between environmental factors with preterm birth that allowed inclusion of covariates listed above that had *p* < 0.05 in crude risk calculations.

To explore the hypothesis that there is a double jeopardy when populations are vulnerable to both social and environmental stressors, we examined SES and race/ethnicity as potential modifiers in the relationship between environmental contaminants and preterm birth. We stratified analyses to examine the relationships between pollution and preterm birth by high and low SES of the census tract the woman lived in. The low SES group consisted of census tracts with below median levels of poverty, education, unemployment and linguistic isolation (Fig. 1, Table 1). We also stratified the analyses by broad race/ethnicity groups: White/non-Hispanic, non-White/non-Hispanic and Hispanic. These stratified analyses compared above versus below median levels of exposure in Fresno County and risk of preterm birth including early preterm birth.

In sensitivity analyses, we explored several alternative analytic decisions. We evaluated the pollutants continuously, both in individual models and a combined model with social factors. We chose more specific phenotypes of preterm birth including early preterm birth (< 34 weeks) and spontaneous preterm (i.e. premature labor or premature rupture of membranes) to restrict to preterm births that were not the consequence of a known cause or indication. We evaluated the raw scores of the exposure indicators (as opposed to the percentiles). Additionally, we mapped preterm birth prevalence across the county to visually observe the geographic variability.

## Results

### Population characteristics

**Fig. 2:**
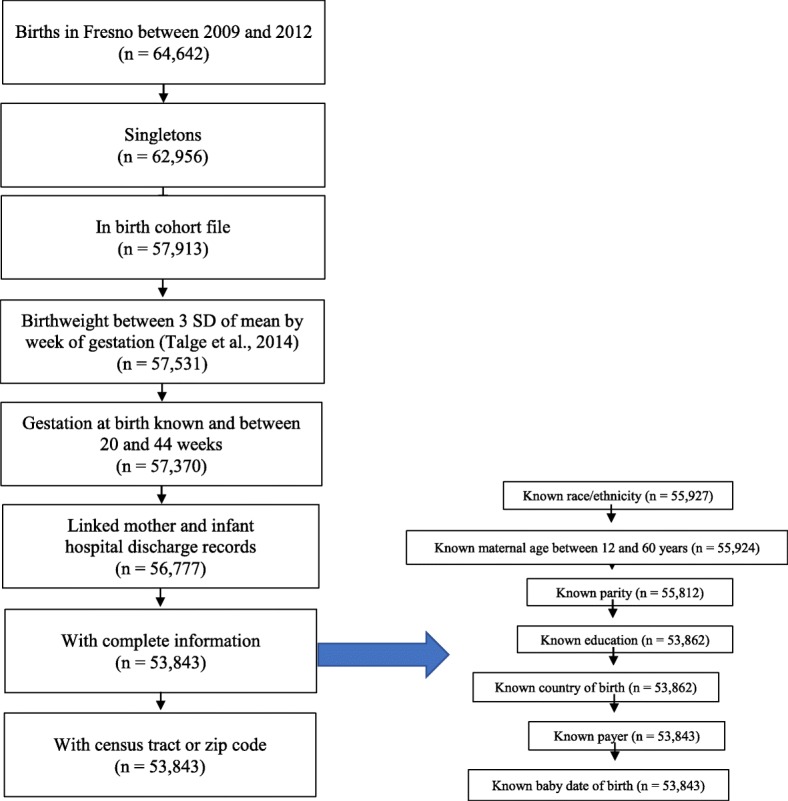
Flow Chart of Our Study Population of Births in Fresno County, California

After applying our exclusion criteria, our final study population included 53,843 births (Fig. 2). Our study population was highly diverse in both race/ethnicity and SES and pollution burden was higher in non-White and low SES areas (Table 2). We did not present cells with less than 16 women (for privacy purposes) nor calculate odds ratios with any cell less than 5. Our population in Fresno County was majority Hispanic (60%), followed by non-Hispanic white (19.7%), Asian (10.5%), and African American (5.8%). One quarter of mothers were born in Mexico (24.5%). More than 30% of the mothers had less than high school education and more than two-thirds of the mothers’ delivery costs were paid by Medi-Cal (California’s Medicaid). The prevalence of preterm birth (< 37 weeks), early preterm birth (< 34 weeks) and spontaneous preterm birth (< 37 weeks and premature rupture of membranes or preterm labor) were 8.5%, 2.1% and 7%, respectively.

**Table 2:** Population characteristics in Fresno County, 2009–2012 (*N* = 53,843)

Population Characteristics	*n* (%)	Pollution Burden Quintile				
		1st	2nd	3rd	4th	5th
Race/ethnicity						
White non-Hispanic	10,620 (19.7)	139 (35.0)	583 (53.5)	2042 (38.3)	4113 (20.0)	3665 (14.0)
Hispanic	32,302 (60.0)	212 (53.4)	357 (32.8)	2186 (41.0)	12,314 (59.7)	17,210 (65.5)
Black	3095 (5.8)	*	17 (1.6)	226 (4.2)	1159 (5.6)	1689 (6.4)
Asian	5675 (10.5)	*	100 (9.2)	512 (9.6)	2141 (10.4)	2899 (11.0)
American Indian/Alaska native	546 (1.0)	19 (4.8)	*	70 (1.3)	201 (1.0)	245 (0.9)
Hawaiian/Pacific Islander	70 (0.1)	*	*	*	40 (0.2)	19 (0.1)
Other race	581 (1.1)	*	*	171 (3.2)	244 (1.2)	155 (0.6)
Two or more races	954 (1.8)	*	*	122 (2.3)	406 (2.0)	395 (1.5)
Infant sex						
Male	27,354 (50.8)	208 (52.4)	569 (52.3)	2675 (50.1)	10,437 (50.6)	13,391 (51.0)
Female	26,489 (49.2)	189 (47.6)	520 (47.8)	2663 (49.9)	10,180 (49.4)	12,886 (49.0)
Maternal age at delivery (years)						
< 18	2263 (4.2)	19 (4.8)	22 (2.0)	132 (2.5)	801 (3.9)	1288 (4.9)
18–34	45,552 (84.6)	340 (84.6)	864 (79.3)	4420 (82.8)	17,506 (84.9)	22,331 (85.0)
> 34	6028 (11.2)	38 (9.6)	203 (18.6)	786 (14.7)	2311 (11.2)	2658 (10.1)
Maternal education (years)						
< 12	16,607 (30.8)	85 (21.4)	132 (12.1)	990 (18.6)	5877 (28.5)	9522 (36.2)
12	15,195 (28.2)	159 (40.1)	232 (21.3)	1275 (23.9)	6063 (29.4)	7456 (28.4)
> 12	22,041 (40.9)	153 (38.5)	725 (66.6)	3073 (57.6)	8678 (42.1)	9299 (35.4)
WIC participant^a^						
Yes	39,404 (73.2)	287 (72.4)	436 (40.0)	2760 (51.7)	15,190 (73.7)	20,723 (79.0)
No	14,439 (26.8)	150 (37.8)	655 (60.2)	2532 (47.4)	5305 (25.7)	5192 (9.8)
Payer for delivery costs						
Private insurance	13,949 (25.9)	150 (37.8)	655 (60.2)	2532 (47.4)	5305 (25.7)	5192 (9.8)
Medi-Cal	39,040 (72.5)	222 (55.9)	399 (36.6)	2730 (51.1)	15,015 (72.8)	20,665 (78.6)
Other government payer	651 (1.2)	*	22 (2.0)	20 (0.4)	36 (0.2)	43 (0.2)
Self-pay	134 (0.3)	*	*	50 (0.9)	230 (1.1)	346 (1.3)
Other payer	2 (0.0)	*	*	*	*	*
No pay	67 (0.1)	*	*	*	31 (0.2)	30 (0.1)
Place of mother’s birth						
United States	35,911 (66.7)	317 (79.9)	825 (75.8)	3881 (72.7)	13,930 (67.6)	16,854 (64.1)
Mexico	13,174 (24.5)	68 (17.1)	121 (11.1)	737 (13.8)	4865 (23.6)	7382 (28.1)
Other	4758 (8.8)	12 (3.0)	143 (13.1)	720 (13.5)	1823 (8.8)	2041 (7.8)
Maternal conditions^b^						
Diabetes, preexisting	565 (1.1)	*	*	33 (0.6)	220 (1.7)	302 (1.2)
Diabetes, gestational	4875 (9.1)	42 (10.6)	86 (7.9)	429 (8.0)	1796 (8.7)	2517 (9.6)
Hypertension, preexisting	915 (1.7)	*	*	103 (1.9)	351 (1.7)	436 (1.7)
Without preeclampsia	649 (1.2)	*	*	75 (1.4)	254 (1.2)	298 (1.1)
With preeclampsia	266 (0.5)	*	*	28 (0.5)	97 (0.5)	138 (0.5)
Hypertension, gestational	3004 (5.6)	*	47 (4.3)	294 (5.5)	1083 (5.3)	1564 (6.0)
Without preeclampsia	1224 (2.3)	*	21 (1.9)	140 (2.6)	424 (2.1)	629 (2.4)
With preeclampsia	1780 (3.3)	*	26 (2.4)	154 (2.9)	659 (3.2)	936 (3.6)
Infection	7402 (13.8)	45 (11.3)	102 (9.4)	562 (10.5)	2805 (13.6)	3789 (14.8)
Anemia	4187 (7.8)	31 (7.8)	73 (6.7)	423 (7.9)	1887 (9.2)	2725 (10.4)
Mental Illness	1231 (2.3)	*	35 (3.2)	155 (2.9)	680 (3.3)	954 (3.6)
Reported Smoking	276 (0.5)	27 (6.8)	89 (8.2)	451 (8.5)	1664 (8.1)	1947 (7.4)
Reported Drug Abuse	1835 (3.4)	*	*	94 (1.8)	423 (2.1)	697 (2.7)
Reported Alcohol Dependence	5147 (9.6)					
Trimester when prenatal care began						
1st	45,632 (84.8)	307 (77.3)	940 (86.3)	4659 (87.3)	17,575 (85.2)	22,032 (83.9)
2nd	4846 (9.0)	66 (16.6)	93 (8.5)	341 (6.4)	1726 (8.4)	2619 (10.0)
3rd	696 (1.3)	*	20 (1.8)	76 (1.4)	246 (1.2)	340 (1.3)
Multiparous sample	35,638	261	708	3354	13,591	17,651
Previous Cesarean-section	9179 (25.8)	78 (29.9)	208 (29.4)	886 (26.4)	3520 (25.9)	4462 (25.3)
Previous Preterm Birth	403 (1.1)	*	*	48 (1.4)	168 (1.2)	178 (1.0)
Interpregnancy Interval ^c^						
< 6 months	2283 (6.4)	21 (8.1)	28 (4.0)	174 (5.2)	853 (6.3)	1207 (6.8)
6–23 months	11,683 (32.8)	76 (29.1)	271 (38.3)	1142 (34.1)	4407 (32.4)	5748 (32.6)
24–59 months	13,671 (38.4)	112 (42.9)	253 (35.7)	1233 (36.8)	5208 (38.3)	6843 (38.8)
> 59 months	5371 (15.1)	34 (13.0)	95 (13.4)	527 (15.7)	2099 (15.4)	2610 (14.8)

*n < 16

^a^WIC Participation – Women, Infants and Children food and nutrition service

^b^Determined by ICD-9 codes in maternal discharge records: preexisting diabetes (ICD-9 code 250 and 648.0), gestational diabetes (648.8), preexisting hypertension (642.0, 642.1, 642.2, 642.7), gestational hypertension (642.3), preeclampsia/eclampsia (642.4, 642.4, 642.6), infection (646.5, 646.6, 647), anemia (648.2), mental illness (648.4)

^c^Number of months between the delivery date of the preceding live birth and the conception date of the index pregnancy

Correlations were moderate between diesel PM, ozone and traffic, ranging from 0.53 to 0.79 (Additional file 1: Appendix 1a). Nitrate and TCE were also moderately correlated (0.62; Additional file 1: Appendix 1b). Summary statistics of each of the indicators by preterm birth status is presented in Table 3. Although many are similar between the two groups, the Exposures score, PM_2.5_, Diesel PM, Toxic Release, Traffic, Drinking Water Score, Cadmium, Nitrate, Uranium, Solid Waste and Pollution Burden Score were all higher among preterm births.

**Table 3:** Descriptive statistics of environmental indicators by preterm birth status in Fresno County, 2009–2012 (*N* = 53,843)

Environmental exposure	Preterm Birth	Full Term Birth
	< 37 weeks (*N* = 4560)	≥37 weeks (*N* = 49,283)
Exposures Score		
Mean (SD)	64.46 (9.83)	63.53 (10.34)
Median (IQR)	65.23 (60.15–70.14)	64.71 (58.94–69.47)
Ozone		
Mean (SD)	0.31 (0.09)	0.31 (0.09)
Median (IQR)	0.32 (0.27–0.37)	0.32 (0.28–0.38)
Pesticides		
Mean (SD)	452.78 (912.72)	477.23 (965.8)
Median (IQR)	10.33 (0.00–505.73)	10.33 (0.00–524.69)
PM_2.5_		
Mean (SD)	14.18 (1.13)	14.09 (1.26)
Median (IQR)	14.28 (13.89–14.53)	14.25 (13.83–14.51)
Diesel PM		
Mean (SD)	25.99 (17.66)	24.92 (17.54)
Median (IQR)	22.96 (7.93–42.94)	20.79 (7.38–41.76)
Toxic Release		
Mean (SD)	3111.81 (9510.54)	2874.46 (9198.78)
Median (IQR)	469.15 (272.01–1109.65)	381.64 (236.93–1037.49)
Traffic		
Mean (SD)	692.44 (471.82)	670.75 (467.61)
Median (IQR)	621.44 (299.40–941.13)	605.60 (267.04–929.61)
Drinking Water		
Mean (SD)	454.91 (114.09)	453.59 (117.42)
Median (IQR)	406.83 (406.83–513.41)	406.83 (406.83–514.09)
Arsenic		
Mean (SD)	1.38 (2.26)	1.40 (2.24)
Median (IQR)	0.70 (0.70–0.84)	0.70 (0.70–0.86)
Cadmium		
Mean (SD)	0.0007 (0.0076)	0.0006 (0.0068)
Median (IQR)	0.00 (0.00–0.00)	0.00 (0.00–0.00)
1,2-Dibromo-3-chloropropane (DBCP)		
Mean (SD)	0.03 (0.02)	0.03 (0.02)
Median (IQR)	0.03 (0.03–0.03)	0.03 (0.03–0.03)
Hexavalent chromium		
Mean (SD)	0.27 (0.63)	0.27 (0.62)
Median (IQR)	0.00 (0.00–0.06)	0.00 (0.00–0.07)
Lead		
Mean (SD)	0.13 (0.40)	0.14 (0.43)
Median (IQR)	0.00 (0.00–0.02)	0.00 (0.00–0.02)
Nitrate		
Mean (SD)	21.36 (7.36)	21.30 (7.68)
Median (IQR)	25.30 (16.74–25.30)	25.30 (16.71–25.30)
Perchlorate		
Mean (SD)	0.06 (0.33)	0.06 (0.32)
Median (IQR)	0.00 (0.00–0.00)	0.00 (0.00–0.00)
Trichloroethylene (TCE)		
Mean (SD)	0.10 (0.07)	0.09 (0.07)
Median (IQR)	0.15 (0.00–0.15)	0.15 (0.00–0.15)
Trihalomethane (THM)		
Mean (SD)	4.53 (9.80)	5.13 (11.22)
Median (IQR)	2.66 (0.96–2.66)	2.66 (0.96–2.66)
Uranium		
Mean (SD)	3.38 (1.75)	3.36 (1.88)
Median (IQR)	3.12 (3.12–3.18)	3.12 (3.12–3.17)
Maximum Contaminant Level (MCL) Violations		
Mean (SD)	0.85 (1.47)	0.85 (1.48)
Median (IQR)	1.00 (0.00–1.00)	0.99 (0.00–1.00)
Total coliform rule (TCR) Violations		
Mean (SD)	0.11 (0.31)	0.11 (0.32)
Median (IQR)	0.00 (0.00–0.00)	0.00 (0.00–0.00)
Environmental Effects Score		
Mean (SD)	24.68 (19.50)	24.77 (19.33)
Median (IQR)	20.15 (8.30–38.25)	20.45 (8.30–38.25)
Cleanup Sites		
Mean (SD)	6.21 (13.05)	6.32 (13.00)
Median (IQR)	1.00 (0.00–8.00)	1.15 (0.00–8.00)
Groundwater Threats		
Mean (SD)	15.23 (18.67)	15.34 (18.78)
Median (IQR)	9.56 (1.50–20.94)	9.56 (1.50–21.00)
Hazardous Waste		
Mean (SD)	0.36 (1.07)	0.36 (1.10)
Median (IQR)	0.05 (0.00–0.21)	0.05 (0.00–0.21)
Imperial Water Bodies		
Mean (SD)	0.47 (1.35)	0.51 (1.36)
Median (IQR)	0.00 (0.00–0.00)	0.00 (0.00–0.00)
Solid Waste		
Mean (SD)	1.45 (2.76)	1.40 (2.76)
Median (IQR)	0.00 (0.00–2.00)	0.00 (0.00–2.00)
Pollution Burden Score		
Mean (SD)	6.51 (1.04)	6.43 (1.07)
Median (IQR)	6.33 (5.83–7.12)	6.24 (5.79–7.09)

Associations between the covariates and preterm birth included hypertension with pre-eclampsia, drug or alcohol abuse and previous preterm birth as maternal factors strongly associated with preterm birth (data not shown). Additionally, Hispanic, African-American and Asian mothers were more likely to have preterm birth compared to white mothers. Mothers with Medi-Cal payer status had higher risk of preterm birth. Additional risk factors for preterm birth included underweight BMI, diabetes, hypertension without pre-eclampsia, infection, anemia, mental illness, previous cesarean delivery, and short (< 6 months) or long (> 59 months) inter-pregnancy interval. Conversely, mothers that participated in WIC were less likely to deliver preterm.

### Association between environmental pollutants and preterm birth

We found that the mothers in the highest quintile of Exposures score were two times as likely to have preterm birth (< 37 weeks), compared to the lowest quintile in the a priori variable adjustment regardless of different statistical adjustment settings (crude and stepwise adjustment, not shown). We also found the highest three quintiles of Pollution Burden score had statistically higher odds of preterm birth (Table 4).

**Table 4:** Crude and adjusted odds ratio of preterm birth across quintiles of CalEnviroScreen indicators and scores in Fresno County, 2009–2012 (*N* = 53,843)

Type of Preterm Birth	< 37 weeks (*N* = 4560)	≥37 weeks (*N* = 49,283)		
Environmental exposure	*N* (%)	*N* (%)	cOR (95% CI)	aOR^a^ (95% CI)
Exposures Score				
0 – 19th percentile	17 (0.4)	380 (0.8)	Reference	Reference
20 – 39th percentile	74 (1.6)	863 (1.8)	1.84 (1.09, 3.12)	1.73 (1.01, 2.97)
40 – 59th percentile	151 (3.3)	1834 (3.7)	1.78 (1.08, 2.93)	1.85 (1.12, 3.06)
60 – 79th percentile	673 (14.8)	8666 (17.6)	1.68 (1.04, 2.72)	1.64 (1.01, 2.65)
80 – 100th percentile	3633 (79.7)	37,428 (76.0)	2.07 (1.28, 3.33)	2.00 (1.25, 3.23)
Ozone				
0 – 19th percentile	†	†	Reference	Reference
20 – 39th percentile	†	66 (0.1)	NC	NC
40 – 59th percentile	178 (3.9)	2100 (4.3)	NC	NC
60 – 79th percentile	631 (13.8)	6659 (13.5)	NC	NC
80 – 100th percentile	3703 (81.2)	39,729 (80.6)	NC	NC
Pesticides				
0 – 19th percentile	1768 (38.8)	19,587 (39.7)	Reference	Reference
20 – 39th percentile	357 (7.8)	3486 (7.1)	1.12 (1.00, 1.26)	1.13 (1.01, 1.26)
40 – 59th percentile	284 (6.2)	3082 (6.3)	1.02 (0.90, 1.16)	1.00 (0.88, 1.14)
60 – 79th percentile	723 (15.9)	7565 (15.4)	1.05 (0.97, 1.15)	1.05 (0.96, 1.15)
80 – 100th percentile	1428 (31.3)	15,563 (31.6)	1.02 (0.95, 1.09)	0.98 (0.92, 1.06)
PM_2.5_				
0 – 19th percentile	20 (0.4)	315 (0.6)	Reference	Reference
20 – 39th percentile	†	†	4.19 (0.56, 31.20)	4.02 (0.53, 30.23)
40 – 59th percentile	38 (0.8)	650 (1.3)	0.93 (0.54, 1.59)	0.89 (0.51, 1.56)
60 – 79th percentile	37 (0.8)	562 (1.1)	1.03 (0.60, 1.78)	1.07 (0.59, 1.94)
80 – 100th percentile	4295 (94.2)	45,804 (92.9)	1.44 (0.93, 2.23)	1.36 (0.88, 2.11)
Diesel Particulate Matter				
0 – 19th percentile	701 (15.4)	8391 (17.0)	Reference	Reference
20 – 39th percentile	609 (13.4)	6616 (13.4)	1.09 (0.98, 1.22)	1.13 (1.02, 1.27)
40 – 59th percentile	566 (12.4)	6582 (13.4)	1.03 (0.92, 1.15)	1.11 (0.99, 1.25)
60 – 79th percentile	738 (16.2)	7690 (15.6)	1.14 (1.02, 1.26)	1.20 (1.08, 1.33)
80 – 100th percentile	1946 (52.7)	20,004 (40.6)	1.15 (1.05, 1.25)	1.16 (1.06, 1.26)
Toxic Release				
0 – 19th percentile	166 (3.6)	1915 (3.9)	Reference	Reference
20 – 39th percentile	466 (10.2)	6073 (12.3)	0.89 (0.75, 1.07)	0.99 (0.82, 1.19)
40 – 59th percentile	2256 (49.5)	25,163 (51.1)	1.03 (0.88, 1.21)	1.10 (0.94, 1.28)
60 – 79th percentile	1049 (23.0)	10,059 (20.4)	1.18 (1.01, 1.39)	1.21 (1.03, 1.42)
80 – 100th percentile	623 (13.7)	6073 (12.3)	1.17 (0.98, 1.38)	1.16 (0.97, 1.37)
Traffic				
0 – 19th percentile	1668 (36.6)	18,996 (38.5)	Reference	Reference
20 – 39th percentile	962 (21.1)	10,572 (21.5)	1.03 (0.95, 1.12)	1.04 (0.96, 1.12)
40 – 59th percentile	982 (21.5)	10,003 (20.3)	1.11 (1.02, 1.20)	1.09 (1.01, 1.18)
60 – 79th percentile	923 (20.2)	9423 (19.1)	1.11 (1.02, 1.20)	1.09 (1.00, 1.18)
80 – 100th percentile	25 (0.6)	289 (0.6)	0.99 (0.66, 1.46)	0.99 (0.67, 1.47)
Drinking Water				
0 – 19th percentile	29 (0.6)	527 (1.1)	Reference	Reference
20 – 39th percentile	†	†	NC	NC
40 – 59th percentile	382 (8.4)	4570 (9.3)	1.48 (1.01, 2.16)	1.50 (1.02, 2.19)
60 – 79th percentile	2800 (61.4)	29,397 (59.7)	1.67 (1.16, 2.40)	1.67 (1.16, 2.41)
80 – 100th percentile	1337 (29.3)	14,677 (29.8)	1.60 (1.11, 2.31)	1.67 (1.15, 2.41)
Arsenic				
0 – 19th percentile	35 (0.8)	397 (0.8)	Reference	Reference
20 – 39th percentile	190 (4.2)	2323 (4.7)	0.93 (0.65, 1.34)	0.91 (0.63, 1.31)
40 – 59th percentile	3288 (72.1)	34,615 (70.2)	1.07 (0.77, 1.49)	1.04 (0.74, 1.45)
60 – 79th percentile	455 (10.0)	5526 (11.2)	0.94 (0.67, 1.32)	0.93 (0.66, 1.31)
80 – 100th percentile	580 (12.7)	6310 (12.8)	1.04 (0.74, 1.46)	0.98 (0.69, 1.38)
Cadmium				
0 – 19th percentile	4336 (95.1)	46,850 (95.1)	Reference	Reference
20 – 39th percentile	†	89 (0.2)	0.86 (0.41, 1.81)	0.86 (0.41, 1.82)
40 – 59th percentile	†	†	NC	NC
60 – 79th percentile	†	†	0.79 (0.11, 5.59)	0.69 (0.10, 4.90)
80 – 100th percentile	216 (4.7)	2330 (4.7)	1.00 (0.87, 1.15)	1.00 (0.87, 1.14)
1,2-Dibromo-3-chloropropane (DBCP)				
0 – 19th percentile	†	†	Reference	Reference
20 – 39th percentile	†	†	NC	NC
40 – 59th percentile	†	†	NC	NC
60 – 79th percentile	216 (4.7)	2314 (4.7)	NC	NC
80 – 100th percentile	4303 (94.4)	46,330 (94.0)	NC	NC
Hexavalent chromium				
0 – 19th percentile	2731 (59.9)	28,808 (58.5)	Reference	Reference
20 – 39th percentile	162 (3.6)	1799 (3.7)	0.95 (0.81, 1.12)	0.99 (0.85, 1.16)
40 – 59th percentile	671 (14.7)	7035 (14.3)	1.01 (0.92, 1.09)	1.00 (0.92, 1.09)
60 – 79th percentile	426 (9.3)	5077 (10.3)	0.89 (0.81, 0.99)	0.89 (0.81, 0.99)
80 – 100th percentile	219 (4.8)	2151 (4.4)	1.07 (0.92, 1.22)	1.06 (0.92, 1.21)
Lead				
0 – 19th percentile	2781 (61.0)	29,538 (59.9)	Reference	Reference
20 – 39th percentile	51 (1.1)	544 (1.1)	1.00 (0.76, 1.31)	1.02 (0.77, 1.34)
40 – 59th percentile	71 (1.6)	869 (1.8)	0.88 (0.69, 1.11)	0.85 (0.67, 1.07)
60 – 79th percentile	772 (16.9)	8534 (17.3)	0.96 (0.89, 1.04)	0.98 (0.90, 1.06)
80 – 100th percentile	873 (19.1)	9686 (19.7)	0.96 (0.89, 1.04)	1.00 (0.93, 1.08)
Nitrate				
0 – 19th percentile	123 (2.7)	1232 (2.5)	Reference	Reference
20 – 39th percentile	62 (1.4)	807 (1.6)	0.79 (0.58, 1.07)	0.77 (0.56, 1.06)
40 – 59th percentile	56 (1.2)	969 (2.0)	0.60 (0.44, 0.83)	0.59 (0.42, 0.81)
60 – 79th percentile	250 (5.5)	2570 (5.2)	0.98 (0.79, 1.21)	1.01 (0.81, 1.27)
80 – 100th percentile	4057 (89.0)	43,593 (88.5)	0.94 (0.78, 1.12)	1.05 (0.88, 1.26)
Perchlorate				
0 – 19th percentile	3928 (86.1)	42,060 (85.3)	Reference	Reference
20 – 39th percentile	23 (0.5)	305 (0.6)	0.82 (0.54, 1.24)	0.88 (0.58, 1.33)
40 – 59th percentile	90 (2.0)	1001 (2.0)	0.97 (0.78, 1.19)	0.99 (0.81, 1.23)
60 – 79th percentile	164 (3.6)	1902 (3.9)	0.93 (0.80, 1.09)	0.97 (0.83, 1.14)
80 – 100th percentile	355 (7.8)	4015 (8.2)	0.95 (0.85, 1.06)	0.97 (0.87, 1.08)
Trichloroethylene (TCE)				
0 – 19th percentile	1244 (27.3)	13,123 (28.7)	Reference	Reference
20 – 39th percentile	†	100 (0.2)	0.24 (0.06, 0.97)	0.27 (0.07, 1.07)
40 – 59th percentile	24 (0.5)	388 (0.8)	0.72 (0.48, 1.08)	0.76 (0.51, 1.14)
60 – 79th percentile	1144 (25.1)	12,506 (25.4)	1.04 (0.96, 1.12)	1.07 (0.98, 1.16)
80 – 100th percentile	2134 (46.8)	22,054 (44.8)	1.09 (1.02, 1.17)	1.09 (1.01, 1.17)
Trihalomethane (THM)				
0 – 19th percentile	4041 (88.6)	42,863 (87.0)	Reference	Reference
20 – 39th percentile	182 (4.0)	2235 (4.5)	0.87 (0.75, 1.01)	0.86 (0.75, 1.00)
40 – 59th percentile	270 (5.9)	3131 (6.4)	0.92 (0.81, 1.04)	1.01 (0.89, 1.15)
60 – 79th percentile	†	112 (0.2)	1.12 (0.64, 1.98)	1.37 (0.78, 2.43)
80 – 100th percentile	55 (1.2)	937 (1.9)	0.64 (0.49, 0.84)	0.62 (0.47, 0.81)
Uranium				
0 – 19th percentile	29 (0.6)	527 (1.1)	Reference	Reference
20 – 39th percentile	310 (6.8)	3787 (7.7)	1.45 (0.99, 2.12)	1.44 (0.98, 2.11)
40 – 59th percentile	118 (2.6)	1614 (3.3)	1.31 (0.87, 1.96)	1.27 (0.85, 1.92)
60 – 79th percentile	291 (6.4)	3061 (6.2)	1.66 (1.14, 2.44)	1.73 (1.18, 2.55)
80 – 100th percentile	3668 (80.4)	38,646 (78.4)	1.66 (1.15, 2.40)	1.68 (1.17, 2.43)
Maximum Contaminant Level (MCL)Violations				
0 – 19th percentile	1028 (22.5)	11,257 (22.8)	Reference	Reference
20 – 39th percentile	†	65 (0.1)	0.18 (0.03, 1.29)	1.20 (0.03, 1.41)
40 – 59th percentile	†	93 (0.2)	0.37 (0.12, 1.16)	0.40 (0.13, 1.25)
60 – 79th percentile	43 (0.9)	634 (1.3)	0.76 (0.56, 1.03)	0.74 (0.54, 1.00)
80 – 100th percentile	3473 (76.2)	37,122 (75.3)	1.02 (0.95, 1.10)	1.01 (0.94, 1.09)
Total coliform rule (TCR) Violations				
0 – 19th percentile	3118 (68.4)	33,671 (68.3)	Reference	Reference
20 – 39th percentile	113 (2.5)	1250 (2.5)	0.98 (0.81, 1.18)	1.00 (0.83, 1.21)
40 – 59th percentile	95 (2.1)	953 (1.9)	1.07 (0.87, 1.31)	1.06 (0.86, 1.30)
60 – 79th percentile	106 (2.3)	1267 (2.6)	0.91 (0.75, 1.11)	0.92 (0.76, 1.13)
80 – 100th percentile	1128 (24.7)	12,142 (24.6)	1.00 (0.94, 1.07)	1.00 (0.93, 1.07)
Environmental Effects Score				
0 – 19th percentile	1725 (37.8)	18,282 (37.1)	Reference	Reference
20 – 39th percentile	946 (20.8)	10,256 (20.8)	0.98 (0.90, 1.06)	0.97 (0.90, 1.06)
40 – 59th percentile	556 (12.2)	6282 (12.8)	0.94 (0.86, 1.04)	0.92 (0.84, 1.01)
60 – 79th percentile	837 (18.4)	9273 (18.8)	0.96 (0.88, 1.04)	0.93 (0.85, 1.01)
80 – 100th percentile	496 (10.9)	5190 (10.5)	1.01 (0.92, 1.12)	0.94 (0.85, 1.05)
Cleanup Sites				
0 – 19th percentile	2512 (55.1)	26,770 (54.3)	Reference	Reference
20 – 39th percentile	537 (11.8)	5832 (11.8)	0.98 (0.90, 1.08)	1.01 (0.92, 1.11)
40 – 59th percentile	561 (12.3)	6472 (13.1)	0.93 (0.85, 1.02)	0.90 (0.82, 0.99)
60 – 79th percentile	486 (10.7)	5066 (10.3)	1.02 (0.93, 1.12)	1.01 (0.91, 1.11)
80 – 100th percentile	464 (10.2)	5143 (10.4)	0.96 (0.87, 1.07)	0.94 (0.85, 1.03)
Groundwater Threats				
0 – 19th percentile	1772 (38.9)	19,140 (38.8)	Reference	Reference
20 – 39th percentile	683 (15.0)	8462 (15.1)	0.99 (0.91, 1.08)	1.00 (0.91, 1.09)
40 – 59th percentile	976 (21.4)	10,124 (20.5)	1.04 (0.96, 1.12)	1.02 (0.95, 1.11)
60 – 79th percentile	652 (14.3)	7472 (15.2)	0.95 (0.87, 1.04)	0.90 (0.82, 0.99)
80 – 100th percentile	477 (10.5)	5085 (10.3)	1.01 (0.91, 1.12)	0.95 (0.86, 1.06)
Hazardous Waste				
0 – 19th percentile	2274 (49.9)	24,740 (50.2)	Reference	Reference
20 – 39th percentile	709 (15.6)	7424 (15.1)	1.04 (0.95, 1.13)	1.01 (0.93, 1.10)
40 – 59th percentile	658 (14.4)	7179 (14.6)	1.00 (0.91, 1.09)	0.98 (0.90, 1.07)
60 – 79th percentile	446 (9.8)	5020 (10.2)	0.97 (0.8, 1.07)	0.95 (0.86, 1.05)
80 – 100th percentile	473 (10.4)	4920 (10.0)	1.04 (0.94, 1.15)	1.01 (0.92, 1.12)
Imperial Water Bodies				
0 – 19th percentile	4031 (88.4)	42,996 (87.2)	Reference	Reference
20 – 39th percentile	286 (6.3)	3410 (6.9)	0.90 (0.80, 1.02)	0.90 (0.80, 1.02)
40 – 59th percentile	162 (3.6)	2011 (4.1)	0.87 (0.74, 1.02)	0.84 (0.72, 0.98)
60 – 79th percentile	38 (0.8)	434 (0.9)	0.94 (0.68, 1.29)	0.88 (0.64, 1.21)
80 – 100th percentile	43 (0.9)	432 (0.9)	1.06 (0.78, 1.43)	0.92 (0.68, 1.24)
Solid Waste				
0 – 19th percentile	2858 (62.7)	31,070 (63.0)	Reference	Reference
20 – 39th percentile	300 (6.6)	3482 (7.1)	0.94 (0.84, 1.06)	0.90 (0.79, 1.01)
40 – 59th percentile	351 (7.7)	3939 (8.0)	0.97 (0.87, 1.09)	0.95 (0.85, 1.06)
60 – 79th percentile	689 (15.1)	7190 (14.6)	1.04 (0.96, 1.13)	1.01 (0.93, 1.10)
80 – 100th percentile	362 (7.9)	3602 (7.3)	1.08 (0.97, 1.21)	1.05 (0.94, 1.17)
Pollution Burden Score				
0 – 19th percentile	17 (0.4)	380 (0.8)	Reference	Reference
20 – 39th percentile	64 (1.4)	1025 (2.1)	1.37 (0.80, 2.34)	1.38 (0.79, 2.40)
40 – 59th percentile	399 (8.8)	4929 (10.0)	1.75 (1.07, 2.84)	1.78 (1.09, 2.88)
60 – 79th percentile	1780 (39.0)	18,838 (38.2)	2.02 (1.25, 3.25)	1.98 (1.23, 3.19)
80 – 100th percentile	2288 (50.2)	23,989 (48.7)	2.03 (1.26, 3.28)	1.98 (1.23, 3.19)

*cOR* crude odds ratio, *aOR* adjusted odds ratio

^a^Adjusted for maternal race/ethnicity, age, education, payment for delivery

^†^*n* < 16

NC not calculated (owing to lack of variability)

We found the highest quintile of drinking water contaminants was associated with higher odds of preterm birth (Table 4), especially spontaneous preterm birth (data not shown). Specifically, uranium concentrations in drinking water was associated with preterm birth and trichloroethylene (TCE) was associated with early preterm birth. Trihalomethanes (THM) concentrations were inversely associated with preterm birth.

The Exposures score, diesel PM and drinking water contaminants were more strongly associated with increased risk of early preterm birth in the low socioeconomic areas compared to the high socioeconomic areas (Table 5). Similar increases were also observed for early preterm birth among the low SES areas compared to high SES areas (Additional file 1: Appendix 3).

**Table 5:** Crude and adjusted* odds ratio of preterm birth comparing above versus below the median of environmental exposure stratified by census tract-level socioeconomic status (SES) in Fresno County, 2009–2012 (*N* = 53,843)

Environmental Exposure	Low SES				High SES			
	< 37 weeks	≥37 weeks			< 37 weeks	≥37 weeks		
	N (%)	N (%)	cOR (95% CI)	aOR* (95% CI)	*N* (%)	*N* (%)	cOR (95% CI)	aOR* (95% CI)
Sample	2455	24,998			2105	24,285		
Exposures Score								
< 50th	924 (37.6)	10,360 (41.4)	Reference	Reference	1172 (55.7)	14,101 (58.1)	Reference	Reference
≥ 50th	1531 (62.4)	14,638 (58.6)	1.16 (1.07, 1.25)	1.16 (1.06, 1.25)	933 (44.3)	10,184 (41.9)	1.09 (1.00, 1.19)	1.07 (0.98, 1.17)
Ozone								
< 50th	1481 (60.4)	15,063 (60.3)	Reference	Reference	777 (36.9)	8752 (36.0)	Reference	Reference
≥ 50th	974 (39.7)	9935 (39.7)	1.00 (0.92, 1.08)	1.00 (0.92, 1.08)	1295 (61.5)	14,916 (61.4)	0.98 (0.90, 1.07)	0.99 (0.90, 1.08)
Pesticides								
< 50th	1019 (41.5)	9872 (39.5)	Reference	Reference	1244 (59.1)	14,691 (60.5)	Reference	Reference
≥ 50th	1436 (58.5)	15,126 (60.5)	0.93 (0.86, 1.00)	0.92 (0.85, 1.00)	861 (40.9)	9594 (39.5)	1.05 (0.97, 1.15)	1.05 (0.97, 1.15)
PM_2.5_								
< 50th	592 (24.1)	6434 (25.7)	Reference	Reference	1425 (67.7)	17,259 (71.1)	Reference	Reference
≥ 50th	1736 (70.7)	17,286 (69.2)	1.08 (0.99, 1.19)	1.07 (0.98, 1.18)	650 (30.9)	6492 (26.7)	1.19 (1.09, 1.31)	1.15 (1.05, 1.26)
Diesel PM								
< 50th	1096 (44.6)	12,166 (48.7)	Reference	Reference	1051 (49.9)	12,587 (51.8)	Reference	Reference
≥ 50th	1359 (55.4)	12,832 (51.3)	1.16 (1.07, 1.25)	1.16 (1.07, 1.25)	1054 (50.1)	11,698 (48.2)	1.07 (0.98, 1.17)	1.04 (0.96, 1.14)
Toxic Release								
< 50th	735 (29.9)	8106 (32.4)	Reference	Reference	1315 (62.5)	16,391 (67.5)	Reference	Reference
≥ 50th	1720 (70.1)	16,892 (67.6)	1.11 (1.02, 1.21)	1.11 (1.02, 1.22)	790 (37.5)	7894 (32.5)	1.22 (1.12, 1.34)	1.18 (1.08, 1.29)
Traffic								
< 50th	1240 (50.5)	13,288 (52.9)	Reference	Reference	972 (46.2)	11,579 (47.7)	Reference	Reference
≥ 50th	1215 (49.5)	11,770 (47.1)	1.09 (1.01, 1.18)	1.09 (1.01, 1.18)	1133 (53.8)	12,706 (52.3)	1.05 (0.97, 1.15)	1.03 (0.95, 1.13)
Drinking Water								
< 50th	143 (5.8)	1863 (7.5)	Reference	Reference	402 (19.1)	4713 (19.4)	Reference	Reference
≥ 50th	2312 (94.2)	23,135 (92.6)	1.27 (1.08, 1.51)	1.29 (1.09, 1.52)	1703 (80.90	19,572 (80.6)	1.02 (0.91, 1.14)	1.00 (0.90, 1.12)
Arsenic								
< 50th	234 (9.5)	2467 (9.9)	Reference	Reference	493 (23.4)	6230 (25.7)	Reference	Reference
≥ 50th	2221 (90.5)	22,531 (90.1)	1.04 (0.91, 1.19)	1.01 (0.88, 1.16)	1612 (76.6)	18,055 (74.4)	1.12 (1.01, 1.24)	1.09 (0.99, 1.21)
Cadmium								
< 50th	0 (0.0)	0 (0.0)	Reference	Reference	0 (0.0)	0 (0.0)	Reference	Reference
≥ 50th	2455 (100.0)	24,998 (100.0)	NC	NC	2105 (100.0)	24,285 (100.0)	NC	NC
1,2-Dibromo-3-chloropropane (DBCP)								
< 50th	979 (39.9)	10,100 (40.4)	Reference	Reference	604 (28.7)	6924 (28.5)	Reference	Reference
≥ 50th	1476 (60.1)	14,898 (59.5)	1.02 (0.94, 1.11)	1.02 (0.94, 1.10)	1472 (69.9)	16,834 (69.3)	1.00 (0.91, 1.10)	1.00 (0.91, 1.10)
Hexavalent Chromium								
< 50th	0 (0.0)	0 (0.0)	Reference	Reference	0 (0.0)	0 (0.0)	Reference	Reference
≥ 50th	2147 (87.5)	21,281 (85.1)	NC	NC	2105 (100.0)	24,285 (100.0)	NC	NC
Lead								
< 50th	0 (0.0)	0 (0.0)	Reference	Reference	0 (0.0)	0 (0.0)	Reference	Reference
≥ 50th	2455 (100.0)	24,998 (100.0)	NC	NC	2105 (100.0)	24,285 (100.0)	NC	NC
Nitrate								
< 50th	1019 (41.5)	10,330 (41.3)	Reference	Reference	1226 (58.2)	14,410 (59.3)	Reference	Reference
≥ 50th	1436 (58.5)	14,668 (58.7)	0.99 (0.92, 1.08)	0.99 (0.92, 1.07)	879 (41.8)	9875 (40.7)	1.04 (0.96, 1.14)	1.03 (0.94, 1.12)
Perchlorate								
< 50th	0 (0.0)	0 (0.0)	Reference	Reference	0 (0.0)	0 (0.0)	Reference	Reference
≥ 50th	2455 (100.0)	24,998 (100.0)	NC	NC	2105 (100.0)	24,285 (100.0)	NC	NC
Trichloroethylene (TCE)								
< 50th	1036 (42.2)	11,114 (44.5)	Reference	Reference	1182 (56.2)	13,607 (56.0)	Reference	Reference
≥ 50th	1419 (57.8)	13,884 (55.5)	1.09 (1.00, 1.18)	1.07 (1.00, 1.17)	923 (43.9)	10,678 (44.0)	1.00 (0.91, 1.09)	0.99 (0.91, 1.08)
Trihalomethane (THM)								
< 50th	1145 (46.6)	11,838 (47.4)	Reference	Reference	859 (40.8)	9593 (39.5)	Reference	Reference
≥ 50th	1310 (53.4)	13,160 (52.6)	1.03 (0.95, 1.11)	1.01 (0.93, 1.09)	1246 (59.2)	14,692 (60.5)	0.95 (0.87, 1.04)	0.96 (0.88, 1.04)
Uranium								
< 50th	487 (19.8)	5535 (22.1)	Reference	Reference	294 (14.0)	3801 (15.7)	Reference	Reference
≥ 50th	1968 (80.2)	19,463 (77.9)	1.15 (1.04, 1.27)	1.14 (1.03, 1.25)	1679 (79.8)	18,9488 (78.0)	1.13 (1.00, 1.28)	1.12 (0.99, 1.27)
Maximum Contaminant Level (MCL)Violations								
< 50th	1030 (42.0)	10,577 (42.3)	Reference	Reference	1207 (57.3)	13,998 (57.6)	Reference	Reference
≥ 50th	1425 (58.0)	14,4211 (57.7)	1.01 (0.94, 1.10)	1.00 (0.92, 1.09)	898 (42.7)	10,287 (42.4)	1.01 (0.93, 1.10)	1.00 (0.92, 1.09)
Total coliform rule (TCR) Violations								
< 50th	0 (0.0)	0 (0.0)	Reference	Reference	0 (0.0)	0 (0.0)	Reference	Reference
≥ 50th	2455 (100.0)	24,998 (100.0)	NC	NC	2105 (100.0)	24,285 (100.0)	NC	NC
Environmental Effects Score								
< 50th	1036 (42.2)	10,073 (40.3)	Reference	Reference	1254 (59.6)	14,381 (59.2)	Reference	Reference
≥ 50th	1419 (57.8)	14,925 (59.7)	0.93 (0.86, 1.01)	0.92 (0.85, 1.00)	851 (40.4)	9904 (40.8)	0.99 (0.90, 1.08)	0.98 (0.89, 1.07)
Cleanup Sites								
< 50th	1168 (47.6)	11,805 (47.2)	Reference	Reference	1139 (54.1)	12,799 (52.7)	Reference	Reference
≥ 50th	1287 (52.4)	13,193 (52.8)	0.99 (0.91, 1.07)	0.97 (0.90, 1.05)	966 (45.9)	11,486 (47.3)	0.95 (0.87, 1.03)	0.95 (0.87, 1.04)
Groundwater Threats								
< 50th	973 (39.6)	9545 (38.2)	Reference	Reference	1290 (61.3)	15,062 (62.0)	Reference	Reference
≥ 50th	1482 (60.4)	15,453 (61.8)	0.95 (0.87, 1.03)	0.93 (0.86, 1.01)	815 (38.7)	9223 (38.)	1.03 (0.94, 1.12)	1.02 (0.94, 1.12)
Hazardous Waste								
< 50th	1023 (41.7)	10,195 (40.8)	Reference	Reference	1240 (58.9)	14,430 (59.4)	Reference	Reference
≥ 50th	1432 (58.3)	14,803 (59.2)	0.97 (0.89, 1.05)	0.98 (0.90, 1.06)	865 (41.1)	9855 (40.6)	1.02 (0.93, 1.11)	0.99 (0.91, 1.09)
Impaired Water Bodies								
< 50th	0 (0.0)	0 (0.0)	Reference	Reference	0 (0.0)	0 (0.0)	Reference	Reference
≥ 50th	2455 (100.0)	24,998 (100.0)	NC	NC	2105 (100.0)	24,285 (100.0)	NC	NC
Solid Waste								
< 50th	0 (0.0)	0 (0.0)	Reference	Reference	0 (0.0)	0 (0.0)	Reference	Reference
≥ 50th	2455 (100.0)	24,998 (100.0)	NC	NC	2105 (100.0)	24,285 (100.0)	NC	NC
Pollution Burden Score								
< 50th	827 (33.7)	8615 (35.5)	Reference	Reference	1380 (65.6)	16,068 (66.2)	Reference	Reference
≥ 50th	1628 (66.3)	16,383 (65.5)	1.03 (0.95, 1.12)	1.04 (0.96, 1.13)	725 (34.4)	8217 (33.8)	1.03 (0.94, 1.12)	1.03 (0.93, 1.11)

*NC* Not Calculated, *cOR* crude odds ratio, *aOR* adjusted odds ratio

^*^Adjusted for maternal race/ethnicity, age, education, payment for delivery

SES defined as “Socioeconomic Factors” score from the CalEnviroScreen, which includes the following variables derived from the US Census American Community Survey: educational attainment, linguistic isolation (households where no one over 14 years of age speaks English very well), poverty and unemployment

We also found the association between diesel PM and preterm birth was slightly higher among non-white and non-Hispanic women, particularly for early preterm birth after adjusting for age, education and payment for delivery costs (Table 6).

**Table 6:** Crude and adjusted odds ratio of preterm birth comparing above versus below the median of environmental exposure stratified by race/ethnicity in Fresno County, 2009–2012 (*N* = 53,843)

Environmental Exposure	White non-Hispanic				Non-White*, Non-Hispanic				Hispanic			
	< 37 weeks	≥37 weeks	cOR (95% CI)	aOR^†^ (95% CI)	< 37 weeks	≥37 weeks	cOR (95% CI)	aOR^†^ (95% CI)	< 37 weeks	≥37 weeks	cOR (95% CI)	aOR^†^ (95% CI)
	N (%)	N (%)			N (%)	N (%)			N (%)	N (%)		
Sample	773	9847			1081	9840			2706	29,596		
Exposures Score												
< 50th	446 (57.7)	5952 (60.4)	Reference	Reference	363 (33.6)	3676 (37.4)	Reference	Reference	1287 (47.6)	14,833 (50.1)	Reference	Reference
≥ 50th	327 (42.3)	3895 (39.6)	1.11 (0.96, 1.28)	1.08 (0.93, 1.24)	718 (66.4)	6164 (62.6)	1.16 (1.02, 1.32)	1.08 (0.94, 1.22)	1419 (52.4)	14,763 (49.9)	1.10 (1.02, 1.18)	1.10 (1.02, 1.19)
Ozone												
< 50th	298 (38.6)	3527 (35.8)	Reference	Reference	467 (43.2)	4402 (44.7)	Reference	Reference	1493 (55.2)	15,886 (53.7)	Reference	Reference
≥ 50th	466 (60.3)	6149 (62.5)	0.90 (0.78, 1.05)	0.90 (0.78, 1.04)	611 (56.5)	5377 (54.6)	1.06 (0.94, 1.20)	1.07 (0.95, 1.21)	1192 (44.1)	13,325 (45.0)	0.96 (0.89, 1.03)	0.97 (0.89, 1.04)
Pesticides												
< 50th	456 (59.0)	5849 (59.4)	Reference	Reference	680 (62.9)	6035 (61.3)	Reference	Reference	1127 (41.7)	12,679 (42.8)	Reference	Reference
≥ 50th	317 (41.0)	3998 (40.6)	1.02 (0.88, 1.17)	1.03 (0.89, 1.19)	401 (37.1)	3805 (38.7)	0.94 (0.83, 1.07)	0.96 (0.84, 1.08)	1579 (58.4)	16,917 (57.2)	1.05 (0.97, 1.13)	1.03 (0.95, 1.11)
PM_2.5_												
< 50th	526 (68.1)	7053 (71.6)	Reference	Reference	502 (46.4)	4926 (50.1)	Reference	Reference	989 (36.6)	11,714 (39.6)	Reference	Reference
≥ 50th	237 (30.7)	2658 (27.0)	1.18 (1.01, 1.38)	1.14 (0.97, 1.33)	558 (51.6)	4627 (47.0)	1.16 (1.03, 1.31)	1.10 (0.97, 1.24)	1591 (58.8)	16,493 (55.7)	1.13 (1.04, 1.22)	1.11 (1.02, 1.20)
Diesel PM												
< 50th	391 (50.6)	5351 (54.3)	Reference	Reference	363 (33.6)	3891 (39.5)	Reference	Reference	1393 (51.5)	15,511 (52.4)	Reference	Reference
≥ 50th	382 (49.4)	4496 (45.7)	1.15 (1.00, 1.32)	1.10 (0.95, 1.27)	718 (66.4)	5949 (60.5)	1.26 (1.11, 1.43)	1.16 (1.02, 1.32)	1313 (48.5)	14,085 (47.6)	1.03 (0.96, 1.12)	1.03 (0.96, 1.12)
Toxic Release												
< 50th	505 (65.3)	6961 (70.7)	Reference	Reference	381 (35.3)	4085 (41.5)	Reference	Reference	1164 (43.0)	13,451 (45.5)	Reference	Reference
≥ 50th	268 (34.7)	2886 (29.3)	1.26 (1.08, 1.46)	1.20 (1.03, 1.40)	700 (64.8)	5755 (58.5)	1.27 (1.12, 1.44)	1.17 (1.03, 1.33)	1542 (57.0)	16,145 (54.6)	1.09 (1.01, 1.18)	1.09 (1.01, 1.17)
Traffic												
< 50th	402 (52.0)	5334 (54.2)	Reference	Reference	378 (35.0)	3960 (40.2)	Reference	Reference	1432 (52.9)	15,513 (52.4)	Reference	Reference
≥ 50th	371 (48.0)	4513 (45.8)	1.08 (0.94, 1.25)	1.04 (0.90, 1.20)	703 (65.0)	5880 (59.8)	1.23 (1.08, 1.39)	1.15 (1.01, 1.31)	1274 (47.1)	14,083 (47.60	0.98 (0.91, 1.06)	0.99 (0.91, 1.07)
Drinking Water												
< 50th	158 (20.4)	1953 (19.8)	Reference	Reference	98 (9.1)	980 (10.0)	Reference	Reference	289 (10.7)	3643 (12.3)	Reference	Reference
≥ 50th	615 (79.6)	7894 (80.2)	0.97 (0.81, 1.15)	0.95 (0.80, 1.13)	983 (90.9)	8860 (90.0)	1.10 (0.89, 1.35)	1.00 (0.81, 1.24)	2417 (89.3)	25,953 (87.7)	1.16 (1.03, 1.31)	1.15 (1.02, 1.30)
Arsenic												
< 50th	225 (29.1)	2843 (28.9)	Reference	Reference	130 (12.0)	1471 (15.0)	Reference	Reference	372 (13.8)	4383 (14.8)	Reference	Reference
≥ 50th	548 (70.9)	7004 (71.3)	0.99 (0.85, 1.16)	0.97 (0.83, 1.14)	951 (88.0)	8369 (85.1)	1.26 (1.05, 1.51)	1.13 (0.94, 1.36)	2334 (86.3)	25,213 (85.2)	1.08 (0.97, 1.21)	1.07 (0.96, 1.19)
Cadmium												
< 50th	0 (0.0)	0 (0.0)	Reference	Reference	0 (0.0)	0 (0.0)	Reference	Reference	0 (0.0)	0 (0.0)	Reference	Reference
≥ 50th	773 (100.0)	9847 (100.0)	NC	NC	1081 (100.0)	9840 (100.0)	NC	NC	2706 (100.0)	29,596 (100.0)	NC	NC
1,2-Dibromo-3-chloropropane (DBCP)												
< 50th	249 (32.2)	3094 (31.4)	Reference	Reference	313 (39.0)	2891 (29.4)	Reference	Reference	1021 (37.7)	11,039 (37.3)	Reference	Reference
≥ 50th	518 (67.0)	6640 (67.4)	0.97 (0.84, 1.13)	0.97 (0.83, 1.13)	766 (70.9)	6909 (70.2)	1.02 (0.90, 1.17)	1.00 (0.87, 1.14)	1664 (61.5)	18,183 (61.4)	0.99 (0.92, 1.07)	1.00 (0.92, 1.08)
Hexavalent Chromium												
< 50th	0 (0.0)	0 (0.0)	Reference	Reference	0 (0.0)	0 (0.0)	Reference	Reference	0 (0.0)	0 (0.0)	Reference	Reference
≥ 50th	773 (100.0)	9847 (100.0)	NC	NC	1081 (100.0)	9840 (100.0)	NC	NC	2706 (100.0)	29,596 (100.0)	NC	NC
Lead												
< 50th	0 (0.0)	0 (0.0)	Reference	Reference	0 (0.0)	0 (0.0)	Reference	Reference	0 (0.0)	0 (0.0)	Reference	Reference
≥ 50th	773 (100.0)	9847 (100.0)	NC	NC	1081 (100.0)	9840 (100.0)	NC	NC	2706 (100.0)	29,596 (100.0)	NC	NC
Nitrate												
< 50th	444 (57.4)	5792 (58.8)	Reference	Reference	430 (39.8)	4435 (45.1)	Reference	Reference	1371 (50.7)	14,513 (49.0)	Reference	Reference
≥ 50th	329 (42.6)	4055 (41.2)	1.05 (0.91, 1.22)	1.02 (0.89, 1.18)	651 (60.2)	5405 (54.9)	1.22 (1.08, 1.37)	1.12 (1.00, 1.27)	1335 (49.3)	15,083 (51.0)	0.94 (0.87, 1.02)	0.94 (0.87, 1.01)
Perchlorate												
< 50th	0 (0.0)	0 (0.0)	Reference	Reference	0 (0.0)	0 (0.0)	Reference	Reference	0 (0.0)	0 (0.0)	Reference	Reference
≥ 50th	773 (100.0)	9847 (100.0)	NC	NC	1081 (100.0)	9840 (100.0)	NC	NC	2706 (100.0)	29,596 (100.0)	NC	NC
Trichloroethylene (TCE)												
< 50th	434 (556.1)	5472 (55.6)	Reference	Reference	387 (35.8)	3949 (40.1)	Reference	Reference	1397 (51.6)	15,300 (51.7)	Reference	Reference
≥ 50th	339 (43.9)	4375 (44.4)	0.98 (0.85, 1.13)	0.95 (0.82, 1.10)	694 (64.2)	5891 (59.9)	1.18 (1.04, 1.34)	1.07 (0.94, 1.22)	1309 (48.4)	14,296 (48.3)	1.00 (0.93, 1.08)	1.00 (0.93, 1.08)
Trihalomethane (THM)												
< 50th	303 (39.2)	3904 (39.7)	Reference	Reference	389 (36.0)	3591 (36.5)	Reference	Reference	1312 (48.5)	13,936 (47.1)	Reference	Reference
≥ 50th	470 (60.8)	5943 (60.4)	1.02 (0.88, 1.18)	1.01 (0.87, 1.16)	692 (64.0)	6249 (63.5)	1.02 (0.90, 1.15)	0.98 (0.86, 1.11)	1394 (51.5)	15,660 (52.9)	0.95 (0.88, 1.02)	0.95 (0.88, 1.02)
Uranium												
< 50th	121 (15.7)	1781 (18.1)	Reference	Reference	130 (12.0)	1304 (13.3)	Reference	Reference	530 (19.6)	6251 (21.1)	Reference	Reference
≥ 50th	584 (75.6)	7293 (74.1)	1.17 (0.96, 1.42)	1.17 (0.96, 1.42)	931 (86.1)	8222 (83.6)	1.12 (0.93, 1.35)	1.07 (0.89, 1.29)	2132 (78.8)	22,896 (77.4)	1.09 (0.99, 1.20)	1.10 (1.00, 1.21)
Maximum Contaminant Level (MCL)Violations												
< 50th	451 (58.3)	5704 (57.9)	Reference	Reference	429 (39.7)	4234 (43.0)	Reference	Reference	1357 (50.2)	14,637 (49.5)	Reference	Reference
≥ 50th	322 (41.7)	4143 (42.1)	0.98 (0.85, 1.14)	0.95 (0.83, 1.11)	652 (60.3)	5606 (57.0)	1.13 (1.00, 1.28)	1.03 (0.91, 1.17)	1349 (49.9)	14,959 (50.5)	0.98 (0.90, 1.05)	0.97 (0.90, 1.04)
Total coliform rule (TCR) Violations												
< 50th	0 (0.0)	0 (0.0)	Reference	Reference	0 (0.0)	0 (0.0)	Reference	Reference	0 (0.0)	0 (0.0)	Reference	Reference
≥ 50th	773 (100.0)	9847 (100.0)	NC	NC	1081 (100.0)	9840 (100.0)	NC	NC	2706 (100.0)	29,596 (100.0)	NC	NC
Environmental Effects Score												
< 50th	444 (57.4)	5779 (58.7)	Reference	Reference	643 (59.5)	5668 (57.6)	Reference	Reference	1203 (44.5)	13,007 (44.0)	Reference	Reference
≥ 50th	329 (42.6)	4068 (41.3)	1.05 (0.91, 1.21)	1.04 (0.90, 1.20)	438 (40.5)	4172 (42.4)	0.93 (0.83, 1.05)	0.92 (0.81, 1.04)	1503 (55.5)	16,589 (56.1)	0.98 (0.91, 1.06)	0.97 (0.89, 1.04)
Cleanup Sites												
< 50th	404 (52.3)	5228 (53.1)	Reference	Reference	584 (54.1)	5144 (52.3)	Reference	Reference	1318 (48.7)	14,232 (48.1)	Reference	Reference
≥ 50th	369 (47.7)	4619 (46.9)	1.03 (0.90, 1.19)	1.03 (0.90, 1.19)	496 (45.9)	4696 (47.7)	0.94 (0.83, 1.05)	0.93 (0.83, 1.05)	1388 (51.3)	15,364 (51.9)	0.98 (0.91, 1.05)	0.97 (0.90, 1.05)
Groundwater Threats												
< 50th	440 (56.9)	5861 (59.5)	Reference	Reference	619 (57.3)	5456 (55.5)	Reference	Reference	1204 (44.5)	13,290 (44.9)	Reference	Reference
≥ 50th	333 (43.1)	3986 (40.5)	1.10 (0.96, 1.27)	1.09 (0.95, 1.26)	462 (42.7)	4384 (44.6)	0.94 (0.83, 1.06)	0.92 (0.81, 1.04)	1502 (55.5)	16,306 (55.1)	1.02 (0.94, 1.10)	1.00 (0.93, 1.08)
Hazardous Waste												
< 50th	437 (56.5)	5951 (60.4)	Reference	Reference	592 (54.8)	5170 (52.5)	Reference	Reference	1234 (45.6)	13,504 (45.6)	Reference	Reference
≥ 50th	336 (43.5)	3896 (39.6)	1.16 (1.01, 1.34)	1.12 (0.97, 1.30)	489 (45.2)	4670 (47.5)	0.92 (0.82, 1.04)	0.90 (0.80, 1.02)	1472 (54.4)	16,092 (54.4)	1.00 (0.93, 1.08)	1.00 (0.92, 1.07)
Impaired Water Bodies												
< 50th	0 (0.0)	0 (0.0)	Reference	Reference	0 (0.0)	0 (0.0)	Reference	Reference	0 (0.0)	0 (0.0)	Reference	Reference
≥ 50th	773 (100.0)	9847 (100.0)	NC	NC	1081 (100.0)	9840 (100.0)	NC	NC	2706 (100.0)	29,596 (100.0)	NC	NC
Solid Waste												
< 50th	0 (0.0)	0 (0.0)	Reference	Reference	0 (0.0)	0 (0.0)	Reference	Reference	0 (0.0)	0 (0.0)	Reference	Reference
≥ 50th	773 (100.0)	9847 (100.0)	NC	NC	1081 (100.0)	9840 (100.0)	NC	NC	2706 (100.0)	29,596 (100.0)	NC	NC
Pollution Burden Score												
< 50th	474 (61.3)	6263 (63.6)	Reference	Reference	515 (47.6)	4827 (49.1)	Reference	Reference	1218 (45.0)	13,593 (45.9)	Reference	Reference
≥ 50th	299 (38.7)	3584 (36.4)	1.09 (0.95, 1.26)	1.08 (0.93, 1.25)	566 (52.4)	5013 (51.0)	1.05 (0.93, 1.19)	1.00 (0.98, 1.12)	1488 (55.0)	16,003 (54.1)	1.03 (0.96, 1.12)	1.03 (0.95, 1.11)

*NC* Not Calculated, *cOR* crude odds ratio, *aOR* adjusted odds ratio

^*^Asian, African-American, Other

^†^Adjusted for maternal race/ethnicity, age, education, payment for delivery

### Sensitivity analyses

In logistic regression models of preterm birth (< 37 weeks gestation) examining one indicator at a time continuously, two pollutant measures were statistically associated with preterm birth: interquartile range increases in PM_2.5_ and Pollution Burden Score were associated with 6% increases in odds of preterm birth after adjustment for education, payer of delivery, maternal age and race/ethnicity. Diesel PM, traffic density and Trichloroethylene concentration (in drinking water) were associated with 26.3% increased odds of early preterm birth (26%, 10%, 16%, respectively, Additional file 1: Appendix 2). The associations were consistent between toxic releases and preterm across all race/ethnicity groups, but highest for white, non-Hispanic early preterm births (Additional file 1: Appendix 4). Pesticides were found to be inversely associated with early preterm birth (data not shown).

When all individual environmental indicators and social factors were included in the same model, PM_2.5_ and unemployment, maternal age > 34, Medi-Cal payer of delivery and African-American race were associated with preterm birth (data not shown). Results examining raw scores were comparable to those of the percentiles.

## Discussion

Overall, the current study found small but consistent associations between pollution exposure and preterm birth in Fresno County. Although many of the individual pollutants were not associated with preterm birth, the cumulative scores were consistently associated with preterm birth, including the Exposures score, drinking water contaminants and Pollution Burden score. Novel exposures, such as the toxic releases from facilities, were identified as a potential contributor to preterm birth in Fresno County. There was an exposure-response of increased risk of preterm birth across quintiles of Pollution Burden scores. Furthermore, the relationship between pollution and preterm birth was stronger among areas with lower SES.

Some risk factors of preterm birth, such as hypertension, have large associations though only affect a small portion of the population. The associations found with pollution were smaller, but may affect a larger portion of births across the population. Pollution may be exacerbating diseases and health issues that lead to preterm birth (e.g.*,* hypertension) [34], or operating directly through toxic exposures (through a variety of possible mechanisms) [35].

The results did not differ considerably when restricted to spontaneous preterm birth. In some cases, results were stronger among the more severe early preterm birth (less than 34 weeks). The drinking water contaminant, THM, was associated with a decrease in preterm birth; however, it can be inversely correlated with other contaminants because it is a disinfection by-product commonly found in metropolitan areas.

Our findings add to the literature on environmental risk factors and preterm birth. For example, in previous studies in CA, we found small but consistent effects of air pollution on risk of preterm birth using air pollution measurements at the geocoded residence [26, 36, 37]. Along with the current study, two additional studies found stronger associations between air pollutants and preterm birth for early preterm birth [26, 37]. Additionally, an interaction was also observed between air pollution and neighborhood SES using three U.S. Census indicators at the block group level (unemployment, poverty, income from public assistance) [26] in our previous study in the Central Valley of California. Compared to previous studies of air pollution with more precise exposure assessment, our current results are likely underestimated owing to non-differential exposure misclassification. The trade-off of the potential measurement errors is the ability to combine multiple exposures and examine cumulative pollution effects.

Consistent with previous work on environmental justice, we observed higher pollution burden among those who were non-White and of lower education and income. Additionally, we found stronger, though not statistically different, associations between some environmental indicators and preterm birth in low SES areas. This is consistent with the concept of ‘double jeopardy’ of environmental and socioeconomic stressors [24]. Further work in this area comparing the entire state of California may be more suitable to demonstrate this occurrence. Overall, there were not considerable differences in the association between pollution and preterm birth between racial/ethnic groups.

Notably, WIC participation, which was associated with high pollution burden and requires low SES, was protective against preterm birth. This is an example of a program that may be having a positive effect on reducing preterm birth in Fresno county. The addition of similar programs, which provide access to supplemental foods, healthcare referrals and nutritional education for pregnant women, may further reduce preterm birth in low-income areas.

Despite the large inclusion of the population, our study did have several limitations. One limitation is the imprecise exposure assessment both geographically and temporally. In some cases, the linkage between the birth records and the census tract were not available and this may have resulted in bias, given changes in census tracts are often a result of population growth. The exposure assessment was at the census tract level and the years were pooled for most data sources. Additionally, the CalEnviroScreen was designed as a screening level tool and does not include specific pollutants or chemical exposures that may be affecting this study population. We examined many indicators of pollution that included nested summary measures, which led to many comparisons. Although we did not adjust for multiple comparisons, we present these results as exploratory. Some women may have had two or possibly more births during this time period (2009–2012); however, we were unable to link them and control for these correlated events. Lastly, we assumed that mothers lived constantly throughout their pregnancy in the maternal residence recorded in the birth certificate without relocating from other regions and did not account for time activity patterns or time spent in other geographical areas.

The CalEnviroScreen is a unique tool devised to identify areas of high pollution burden and vulnerable populations and has the benefit of informing epidemiologic studies. Strengths of this study include our ability to include a large set of pollution indicators both individually and cumulatively across a broad geographic area. Additionally, we were able to include all singleton births in Fresno County with detailed demographic and medical information from medical discharge records. Further, our results find a stronger association with the Exposures score, which makes sense as this score consists of monitoring data that is likely to be more representative of actual exposures in the population.

## Conclusion

Our study provides an initial investigation of the CalEnviroScreen as an epidemiologic tool to help elucidate a host of environmental and social factors that contribute to preterm birth. As a screening tool designed to discern communities that assume disproportionate environmental burdens in California, the CalEnviroScreen provides data for environmental justice research. Future studies could expand to the entire state of California and aim to include additional sources of data such as biomonitoring and genomics that could confirm exposure levels and identify pathways by which environmental pollutants contribute to preterm birth.

## Additional file

Additional file 1: **Appendix 1a.** Pearson Correlation Matrix of Exposures from CalEnviroScreen 2.0. **Appendix 1b.** Correlation Matrix of Drinking Water Contaminants from CalEnviroScreen 2.0. **Appendix 2.** Crude and adjusted relative risk of early (< 34 weeks) preterm birth across quintiles of CalEnviroScreen indicators and scores in Fresno County, 2009–2012 (*N* = 50,413). **Appendix 3.** Crude and adjusted relative risk of early (< 34 weeks) preterm birth comparing above versus below the median of environmental exposure by census tract-level socioeconomic status (SES) in Fresno County, 2009–2012 (*N* = 50,413). **Appendix 4.** Crude and adjusted relative risk of early (< 34 weeks) preterm birth comparing above versus below the median of environmental exposure by race/ethnicity in Fresno County, 2009–2012 (*N* = 50,413). (DOCX 88 kb)

## Abbreviations

BMI: Body mass index
CA: California
OSHPD: Office of Statewide Health Planning and Development
PM_2.5_: Particulate matter < 2.5 μm in aerodynamic diameter
SES: Socioeconomic status
