# Supplementary material for: Environmental pollution and social factors as contributors to preterm birth in Fresno County
Source: Environ Health. 2018 Aug 29;17:70. doi: 10.1186/s12940-018-0414-x (PMC6114053; doi:10.1186/s12940-018-0414-x)
Supplement: Supplementary file 1 — Appendix 1a. Pearson Correlation Matrix of Exposures from CalEnviroScreen 2.0. Appendix 1b. Correlation Matrix of Drinking Water Contaminants from CalEnviroScreen 2.0. Appendix 2. Crude and adjusted relative risk of early (< 34 weeks) preterm birth across quintiles of CalEnviroScreen indicators and scores in Fresno County, 2009–2012 (N = 50,413). Appendix 3. Crude and adjusted relative risk of early (< 34 weeks) preterm birth comparing above versus below the median of environmental exposure by census tract-level socioeconomic status (SES) in Fresno County, 2009–2012 (N = 50,413). Appendix 4. Crude and adjusted relative risk of early (< 34 weeks) preterm birth comparing above versus below the median of environmental exposure by race/ethnicity in Fresno County, 2009–2012 (N = 50,413). (DOCX 88 kb) [file 12940_2018_414_MOESM1_ESM.docx]

## Appendix 1a. Pearson Correlation Matrix of Exposures from CalEnviroScreen 2.0

|  | Exposures | Ozone | Pesticides | PM_2.5_ | Diesel PM | Toxic release | Traffic | Drinking water | Environmental Effects | Cleanup sites | Groundwater threats | Hazardous waste | Impaired Water Bodies | Solid waste | Pollution burden |
| --- | --- | --- | --- | --- | --- | --- | --- | --- | --- | --- | --- | --- | --- | --- | --- |
| Exposures | 1.00 | **0.33** | **-0.14** | **0.61** | **0.60** | **0.28** | **0.57** | **0.30** | **0.06** | **0.04** | **-0.11** | **0.10** | **-0.46** | -0.02 | **0.82** |
| Ozone |  | 1.00 | **-0.50** | **0.16** | **0.63** | **-0.12** | **0.53** | **-0.29** | **-0.51** | **-0.18** | **-0.52** | **-0.08** | **-0.53** | **-0.46** | **-0.04** |
| Pesticides |  |  | 1.00 | **-0.05** | **-0.46** | **-0.09** | **-0.36** | **0.26** | **0.32** | **-0.02** | **0.25** | **0.29** | **0.25** | **0.27** | **0.06** |
| PM_2.5_ |  |  |  | 1.00 | **0.17** | **0.14** | **0.14** | **0.13** | **0.12** | **0.12** | *-0.01* | **0.14** | **-0.29** | **0.08** | **0.54** |
| Diesel Particulate Matter (PM) |  |  |  |  | 1.00 | **0.18** | **0.79** | **-0.23** | **-0.36** | **-0.04** | **-0.28** | **-0.11** | **-0.40** | **-0.36** | **0.30** |
| Toxic release |  |  |  |  |  | 1.00 | **0.04** | **-0.06** | **0.37** | **0.73** | **0.34** | **0.10** | **-0.11** | **0.21** | **0.46** |
| Traffic |  |  |  |  |  |  | 1.00 | **-0.07** | **-0.27** | **-0.04** | **-0.22** | 0.00 | **-0.31** | **-0.24** | **0.33** |
| Drinking water |  |  |  |  |  |  |  | 1.00 | **0.13** | **-0.05** | **0.07** | **0.06** | **0.05** | **0.35** | **0.33** |
| Environmental Effects |  |  |  |  |  |  |  |  | 1.00 | **0.62** | **0.79** | **0.35** | **0.35** | **0.62** | **0.58** |
| Cleanup sites |  |  |  |  |  |  |  |  |  | 1.00 | **0.53** | **0.55** | **0.22** | **-0.02** | **0.39** |
| Groundwater threats |  |  |  | *Italics = p < 0.05* | | |  |  |  |  | 1.00 | **0.39** | **0.25** | **0.45** | **0.37** |
| Hazardous waste |  |  |  | Underlined = p < 0.01 | | |  |  |  |  |  | 1.00 | **-0.06** | **0.30** | **0.29** |
| Impaired Water Bodies |  |  |  | **Bold = p < 0.0001** | | |  |  |  |  |  |  | 1.00 | **0.16** | **-0.19** |
| Solid waste |  |  |  |  |  |  |  |  |  |  |  |  |  | 1.00 | **0.32** |
| Pollution burden |  |  |  |  |  |  |  |  |  |  |  |  |  |  | 1.00 |

## Appendix 1b. Correlation Matrix of Drinking Water Contaminants from CalEnviroScreen 2.0

|  | Drinking water | Arsenic | Cadmium | DBCP | Hex Chrom | Lead | Nitrate | Perchlorate | TCE | THM | Uranium | MCL Violations | TCR Violations |
| --- | --- | --- | --- | --- | --- | --- | --- | --- | --- | --- | --- | --- | --- |
| Drinking water | 1.00 | **0.22** | **0.16** | **0.08** | **0.36** | **0.15** | **-0.13** | 0.00 | **-0.08** | **-0.28** | **0.40** | **0.12** | **0.27** |
| Arsenic |  | 1.00 | **0.03** | **-0.14** | **0.34** | **0.18** | **-0.56** | **-0.10** | **-0.39** | **-0.07** | **0.07** | **0.67** | **0.16** |
| Cadmium |  |  | 1.00 | **0.30** | **0.14** | -0.01 | **-0.10** | 0.00 | **-0.02** | **-0.04** | **0.05** | **-0.04** | **0.02** |
| 1,2-Dibromo-3-chloropropane (DBCP) |  |  |  | 1.00 | **-0.03** | **-0.07** | **0.26** | **-0.02** | **-0.04** | **-0.10** | **-0.08** | **-0.12** | **-0.14** |
| Hexavalent chromium |  |  |  |  | 1.00 | **-0.02** | **-0.53** | **-0.04** | **-0.59** | **-0.21** | **0.31** | **-0.13** | **0.31** |
| Lead |  |  |  |  |  | 1.00 | **-0.24** | **0.06** | **-0.38** | **0.33** | **0.45** | **0.07** | **0.09** |
| Nitrate |  |  |  |  |  |  | 1.00 | **-0.05** | **0.62** | **-0.33** | **-0.12** | **-0.20** | **-0.24** |
| Perchlorate |  |  |  |  |  |  |  | 1.00 | **-0.23** | **-0.08** | **0.27** | **-0.10** | **0.43** |
| Trichloroethylene (TCE) |  | *Italics = p < 0.05* | | | |  |  |  | 1.00 | **-0.28** | **-0.17** | **0.10** | **-0.30** |
| Trichloroethylene (THM) |  | Underlined = p < 0.01 | | | |  |  |  |  | 1.00 | **-0.16** | **0.16** | **-0.13** |
| Uranium |  | **Bold = p < 0.0001** | | | |  |  |  |  |  | 1.00 | **-0.12** | **0.17** |
| Maximum Contaminant Level (MCL) Violations |  |  |  |  |  |  |  |  |  |  |  | 1.00 | 0.00 |
| Total coliform rule (TCR) Violations |  |  |  |  |  |  |  |  |  |  |  |  | 1.00 |

## Appendix 2. Crude and adjusted relative risk of early (<34 weeks) preterm birth across quintiles of CalEnviroScreen indicators and scores in Fresno County, 2009-2012 (N=50,413)

| **Type of Preterm Birth** | **<34 weeks**  **(N=1,130)** | **≥37 weeks**  **(N=49,283)** |  |  |
| --- | --- | --- | --- | --- |
| **Environmental exposure** | **N (%)** | **N (%)** | **cRR (95% CI)** | **aRR^*^ (95% CI)** |
| Exposures Score |  |  |  |  |
| 0 – 19^th^ percentile | ^†^ | 380 (0.8) | Reference | Reference |
| 20 – 39^th^ percentile | 23 (2.0) | 863 (1.8) | 2.49 (0.86, 7.21) | 2.36 (0.81, 6.92) |
| 40 – 59^th^ percentile | 32 (2.8) | 1,834 (3.7) | 1.65 (0.58, 4.66) | 1.73 (0.61, 4.92) |
| 60 – 79^th^ percentile | 144 (12.7) | 8,666 (17.6) | 1.57 (0.58, 4.24) | 1.59 (0.59, 4.29) |
| 80 – 100^th^ percentile | 927 (82.0) | 37,428 (76.0) | 2.32 (0.97, 6.20) | 2.30 (0.86, 6.13) |
| Ozone |  |  |  |  |
| 0 – 19^th^ percentile | ^†^ | ^†^ | Reference | Reference |
| 20 – 39^th^ percentile | ^†^ | 66 (0.1) | NC | NC |
| 40 – 59^th^ percentile | 38 (3.4) | 2,100 (4.3) | NC | NC |
| 60 – 79^th^ percentile | 136 (12.0) | 6,659 (13.5) | NC | NC |
| 80 – 100^th^ percentile | 944 (83.50 | 39,729 (80.6) | NC | NC |
| Pesticides |  |  |  |  |
| 0 – 19^th^ percentile | 454 (40.2) | 19,587 (39.7) | Reference | Reference |
| 20 – 39^th^ percentile | 95 (8.4) | 3,486 (7.1) | 1.17 (0.94, 1.46) | 1.18 (0.94, 1.47) |
| 40 – 59^th^ percentile | 79 (7.0) | 3,082 (6.3) | 1.10 (0.87, 1.40) | 1.09 (0.86, 1.38) |
| 60 – 79^th^ percentile | 190 (16.8) | 7,565 (15.4) | 1.08 (0.91, 1.28) | 1.09 (0.92, 1.29) |
| 80 – 100^th^ percentile | 312 (27.6) | 15,563 (31.6) | 0.87 (0.75, 1.00) | 0.84 (0.72, 0.97) |
| PM_2.5_ |  |  |  |  |
| 0 – 19^th^ percentile | ^†^ | 315 (0.6) | Reference | Reference |
| 20 – 39^th^ percentile | ^†^ | ^†^ | NC | NC |
| 40 – 59^th^ percentile | ^†^ | 650 (1.3) | 0.55 (0.21, 1.43) | 0.60 (0.22, 1.61) |
| 60 – 79^th^ percentile | ^†^ | 562 (1.1) | 0.64 (0.25, 1.65) | 0.67 (0.23, 1.95) |
| 80 – 100^th^ percentile | 1,069 (94.6) | 45,804 (92.9) | 0.92 (0.46, 1.85) | 0.86 (0.43, 1.72) |
| Diesel Particulate Matter |  |  |  |  |
| 0 – 19^th^ percentile | 155 (13.7) | 8,391 (17.0) | Reference | Reference |
| 20 – 39^th^ percentile | 129 (11.4) | 6,616 (13.4) | 1.05 (0.83, 1.33) | 1.11 (0.88, 1.41) |
| 40 – 59^th^ percentile | 123 (10.9) | 6,582 (13.4) | 1.01 (0.80, 1.28) | 1.10 (0.86, 1.42) |
| 60 – 79^th^ percentile | 173 (15.3) | 7,690 (15.6) | 1.21 (0.98, 1.51) | 1.28 (1.02, 1.59) |
| 80 – 100^th^ percentile | 550 (48.7) | 20,004 (40.6) | 1.48 (1.23, 1.76) | 1.49 (1.24, 1.78) |
| Toxic Release |  |  |  |  |
| 0 – 19^th^ percentile | 39 (3.5) | 1,915 (3.9) | Reference | Reference |
| 20 – 39^th^ percentile | 99 (8.8) | 6,073 (12.3) | 0.80 (0.55, 1.16) | 0.96 (0.65, 1.42) |
| 40 – 59^th^ percentile | 549 (48.6) | 25,163 (51.1) | 1.07 (0.88, 1.48) | 1.15 (0.83, 1.60) |
| 60 – 79^th^ percentile | 284 (25.1) | 10,059 (20.4) | 1.38 (0.98, 1.92) | 1.41 (1.00, 1.97) |
| 80 – 100^th^ percentile | 159 (14.1) | 6,073 (12.3) | 1.28 (0.90, 1.81) | 1.24 (0.88, 1.77) |
| Traffic |  |  |  |  |
| 0 – 19^th^ percentile | 381 (33.7) | 18,996 (38.5) | Reference | Reference |
| 20 – 39^th^ percentile | 218 (19.3) | 10,572 (21.5) | 1.03 (0.87, 1.21) | 1.02 (0.86, 1.21) |
| 40 – 59^th^ percentile | 283 (25.0) | 10,003 (20.3) | 1.40 (1.20, 1.63) | 1.37 (1.17, 1.60) |
| 60 – 79^th^ percentile | 245 (21.7) | 9,423 (19.1) | 1.29 (1.10, 1.51) | 1.23 (1.05, 1.45) |
| 80 – 100^th^ percentile | ^†^ | 289 (0.6) | 0.52 (0.17, 1.63) | 0.50 (0.16, 1.56) |
| Drinking Water |  |  |  |  |
| 0 – 19^th^ percentile | ^†^ | 527 (1.1) | Reference | Reference |
| 20 – 39^th^ percentile | ^†^ | ^†^ | NC | NC |
| 40 – 59^th^ percentile | 88 (7.8) | 4,570 (9.3) | 1.13 (0.57, 2.23) | 1.27 (0.63, 2.58) |
| 60 – 79^th^ percentile | 719 (63.6) | 29,397 (59.7) | 1.42 (0.74, 2.74) | 1.46 (0.76, 2.83) |
| 80 – 100^th^ percentile | 314 (27.8) | 14,677 (29.8) | 1.25 (0.64, 2.42) | 1.43 (0.73, 2.80) |
| *Arsenic* |  |  |  |  |
| 0 – 19^th^ percentile | ^†^ | 397 (0.8) | Reference | Reference |
| 20 – 39^th^ percentile | 40 (3.5) | 2,323 (4.7) | 1.36 (0.54, 3.45) | 1.27 (0.49, 3.27) |
| 40 – 59^th^ percentile | 862 (76.3) | 34,615 (70.2) | 1.95 (0.81, 4.71) | 1.85 (0.77, 4.46) |
| 60 – 79^th^ percentile | 103 (9.1) | 5,526 (11.2) | 1.47 (0.60, 3.61) | 1.52 (0.62, 3.74) |
| 80 – 100^th^ percentile | 120 (10.6) | 6,310 (12.8) | 1.50 (0.61, 3.67 | 1.35 (0.55, 3.31) |
| *Cadmium* |  |  |  |  |
| 0 – 19^th^ percentile | 1,083 (95.8) | 46,850 (95.1) | Reference | Reference |
| 20 – 39^th^ percentile | ^†^ | 89 (0.2) | 0.97 (0.24, 3.89) | 0.98 (0.25, 3.93) |
| 40 – 59^th^ percentile | ^†^ | ^†^ | NC | NC |
| 60 – 79^th^ percentile | ^†^ | ^†^ | 2.95 (0.42, 20.97) | 2.15 (0.30, 15.35) |
| 80 – 100^th^ percentile | 44 (3.9) | 2,330 (4.7) | 0.82 (0.61, 1.11) | 0.82 (0.61, 1.11) |
| *1,2-Dibromo-3-chloropropane (DBCP)* | |  |  |  |
| 0 – 19^th^ percentile | ^†^ | ^†^ | Reference | Reference |
| 20 – 39^th^ percentile | ^†^ | ^†^ | NC | NC |
| 40 – 59^th^ percentile | ^†^ | ^†^ | NC | NC |
| 60 – 79^th^ percentile | 47 (4.2) | 2,314 (4.7) | NC | NC |
| 80 – 100^th^ percentile | 1,074 (95.0) | 46,330 (94.0) | NC | NC |
| *Hexavalent chromium* |  |  |  |  |
| 0 – 19^th^ percentile | 709 (62.7) | 28,808 (58.5) | Reference | Reference |
| 20 – 39^th^ percentile | 36 (3.2) | 1,799 (3.7) | 0.81 (0.58, 1.14) | 0.87 (0.62, 1.22) |
| 40 – 59^th^ percentile | 170 (15.0) | 7,035 (14.3) | 0.98 (0.83, 1.16) | 0.97 (0.82, 1.15) |
| 60 – 79^th^ percentile | 92 (8.1) | 5,077 (10.3) | 0.74 (0.60, 0.92) | 0.74 (0.60, 0.93) |
| 80 – 100^th^ percentile | 48 (4.3) | 2,151 (4.4) | 0.91 (0.68, 1.22) | 0.90 (0.67, 1.21) |
| *Lead* |  |  |  |  |
| 0 – 19^th^ percentile | 723 (64.0) | 29,538 (59.9) | Reference | Reference |
| 20 – 39^th^ percentile | ^†^ | 544 (1.1) | 0.98 (0.56, 1.69) | 1.01 (0.59, 1.75) |
| 40 – 59^th^ percentile | ^†^ | 869 (1.8) | 0.71 (0.43, 1.18) | 0.68 (0.41, 1.14) |
| 60 – 79^th^ percentile | 175 (15.5) | 8,534 (17.3) | 0.84 (0.71, 0.99) | 0.86 (0.73, 1.02) |
| 80 – 100^th^ percentile | 204 (18.1) | 9,686 (19.7) | 0.86 (0.74, 1.01) | 0.91 (0.78, 1.07) |
| *Nitrate* |  |  |  |  |
| 0 – 19^th^ percentile | 26 (2.3) | 1,232 (2.5) | Reference | Reference |
| 20 – 39^th^ percentile | ^†^ | 807 (1.6) | 0.71 (0.36, 1.41) | 0.66 (0.33, 1.32) |
| 40 – 59^th^ percentile | ^†^ | 969 (2.0) | 0.74 (0.39, 1.39) | 0.66 (0.34, 1.26) |
| 60 – 79^th^ percentile | 55 (4.9) | 2,570 (5.2) | 1.01 (0.64, 1.62) | 1.13 (0.69, 1.86) |
| 80 – 100^th^ percentile | 1,022 (90.4) | 43,593 (88.5) | 1.11 (0.75, 1.64) | 1.29 (0.87, 1.91) |
| *Perchlorate* |  |  |  |  |
| 0 – 19^th^ percentile | 999 (88.4) | 42,060 (85.3) | Reference | Reference |
| 20 – 39^th^ percentile | ^†^ | 305 (0.6) | 0.83 (0.37, 1.86) | 0.92 (0.41, 2.06) |
| 40 – 59^th^ percentile | ^†^ | 1,001 (2.0) | 0.55 (0.32, 0.96) | 0.58 (0.33, 1.00) |
| 60 – 79^th^ percentile | 38 (3.4) | 1,902 (3.9) | 0.84 (0.61, 1.17) | 0.91 (0.67, 1.26) |
| 80 – 100^th^ percentile | 74 (6.6) | 4,015 (8.2) | 0.78 (0.62, 0.99) | 0.81 (0.64, 1.02) |
| *Trichloroethylene (TCE)* | |  |  |  |
| 0 – 19^th^ percentile | 264 (23.4) | 13,123 (28.7) | Reference | Reference |
| 20 – 39^th^ percentile | ^†^ | 100 (0.2) | 0.54 (0.08, 3.84) | 0.63 (0.09, 4.54) |
| 40 – 59^th^ percentile | ^†^ | 388 (0.8) | 0.97 (0.46, 2.05) | 1.04 (0.49, 2.21) |
| 60 – 79^th^ percentile | 297 (26.3) | 12,506 (25.4) | 1.26 (1.07, 1.49) | 1.30 (1.10, 1.54) |
| 80 – 100^th^ percentile | 561 (49.7) | 22,054 (44.8) | 1.35 (1.17, 1.56) | 1.35 (1.16, 1.56) |
| *Trichloroethylene (THM)* | |  |  |  |
| 0 – 19^th^ percentile | 1,004 (88.9) | 42,863 (87.0) | Reference | Reference |
| 20 – 39^th^ percentile | 45 (4.0) | 2,235 (4.5) | 0.86 (0.64, 1.16) | 0.85 (0.63, 1.15) |
| 40 – 59^th^ percentile | 66 (5.8) | 3,131 (6.4) | 0.90 (0.70, 1.16) | 1.04 (0.81, 1.34) |
| 60 – 79^th^ percentile | ^†^ | 112 (0.2) | NC | NC |
| 80 – 100^th^ percentile | ^†^ | 937 (1.9) | 0.69 (0.41, 1.15) | 0.64 (0.38, 1.07) |
| *Uranium* |  |  |  |  |
| 0 – 19^th^ percentile | ^†^ | 527 (1.1) | Reference | Reference |
| 20 – 39^th^ percentile | 66 (5.8) | 3,787 (7.7) | 1.02 (0.51, 2.05) | 1.13 (0.55, 2.33) |
| 40 – 59^th^ percentile | 24 (2.1) | 1,614 (3.3) | 0.87 (0.41, 1.88) | 0.92 (0.42, 2.00) |
| 60 – 79^th^ percentile | 63 (5.6) | 3,061 (6.2) | 1.20 (0.60, 2.41) | 1.32 (0.65, 2.69) |
| 80 – 100^th^ percentile | 944 (83.5) | 38,646 (78.4) | 1.42 (0.74, 2.74) | 1.51 (0.78, 2.92) |
| *Maximum Contaminant Level (MCL) Violations* | | |  |  |
| 0 – 19^th^ percentile | 227 (20.1) | 11,257 (22.8) | Reference | Reference |
| 20 – 39^th^ percentile | ^†^ | 65 (0.1) | NC | NC |
| 40 – 59^th^ percentile | ^†^ | 93 (0.2) | 0.54 (0.08, 3.84) | 0.59 (0.08, 4.20) |
| 60 – 79^th^ percentile | ^†^ | 634 (1.3) | 0.63 (0.31, 1.28) | 0.60 (0.30, 1.22) |
| 80 – 100^th^ percentile | 894 (79.1) | 37,122 (75.3) | 1.19 (1.02, 1.38) | 1.17 (1.01, 1.35) |
| *Total coliform rule (TCR) Violations* | |  |  |  |
| 0 – 19^th^ percentile | 793 (70.2) | 33,671 (68.3) | Reference | Reference |
| 20 – 39^th^ percentile | 25 (2.2) | 1,250 (2.5) | 0.85 (0.57, 1.27) | 0.88 (0.59, 1.31) |
| 40 – 59^th^ percentile | 26 (2.3) | 953 (1.9) | 1.15 (0.78, 1.71) | 1.15 (0.78, 1.69) |
| 60 – 79^th^ percentile | 18 (1.6) | 1,267 (2.6) | 0.61 (0.38, 0.97) | 0.63 (0.39, 1.00) |
| 80 – 100^th^ percentile | 268 (23.7) | 12,142 (24.6) | 0.94 (0.82, 1.08) | 0.94 (0.81, 1.07) |
| Environmental Effects Score |  |  |  |  |
| 0 – 19^th^ percentile | 451 (39.9) | 18,282 (37.1) | Reference | Reference |
| 20 – 39^th^ percentile | 224 (19.8) | 10,256 (20.8) | 0.89 (0.76, 1.04) | 0.88 (0.75, 1.04) |
| 40 – 59^th^ percentile | 137 (12.1) | 6,282 (12.8) | 0.89 (0.73, 1.07) | 0.86 (0.71, 1.04) |
| 60 – 79^th^ percentile | 212 (18.8) | 9,273 (18.8) | 0.93 (0.79, 1.09) | 0.90 (0.76, 1.06) |
| 80 – 100^th^ percentile | 106 (9.4) | 5,190 (10.5) | 0.83 (0.67, 1.03) | 0.77 (0.62, 0.95) |
| Cleanup Sites |  |  |  |  |
| 0 – 19^th^ percentile | 630 (55.8) | 26,770 (54.3) | Reference | Reference |
| 20 – 39^th^ percentile | 146 (12.9) | 5,832 (11.8) | 1.06 (0.89, 1.27) | 1.11 (0.93, 1.33) |
| 40 – 59^th^ percentile | 124 (11.0) | 6,472 (13.1) | 0.82 (0.67, 0.99) | 0.79 (0.65, 0.95) |
| 60 – 79^th^ percentile | 116 (10.3) | 5,066 (10.3) | 0.97 (0.80, 1.19) | 0.95 (0.78, 1.16) |
| 80 – 100^th^ percentile | 114 (10.1) | 5,143 (10.4) | 0.94 (0.77, 1.15) | 0.91 (0.74, 1.11) |
| Groundwater Threats |  |  |  |  |
| 0 – 19^th^ percentile | 428 (37.9) | 19,140 (38.8) | Reference | Reference |
| 20 – 39^th^ percentile | 179 (15.8) | 8,462 (15.1) | 1.07 (0.90, 1.28) | 1.09 (0.91, 1.30) |
| 40 – 59^th^ percentile | 250 (22.1) | 10,124 (20.5) | 1.10 (0.94, 1.29) | 1.08 (0.92, 1.26) |
| 60 – 79^th^ percentile | 163 (14.4) | 7,472 (15.2) | 0.98 (0.82, 1.17) | 0.92 (0.77, 1.10) |
| 80 – 100^th^ percentile | 110 (9.7) | 5,085 (10.3) | 0.97 (0.79, 1.19) | 0.92 (0.74, 1.13) |
| Hazardous Waste |  |  |  |  |
| 0 – 19^th^ percentile | 551 (48.8) | 24,740 (50.2) | Reference | Reference |
| 20 – 39^th^ percentile | 181 (16.0) | 7,424 (15.1) | 1.09 (0.92, 1.29) | 1.06 (0.89, 1.25) |
| 40 – 59^th^ percentile | 165 (14.6) | 7,179 (14.6) | 1.03 (0.87, 1.23) | 1.00 (0.84, 1.19) |
| 60 – 79^th^ percentile | 121 (10.7) | 5,020 (10.2) | 1.08 (0.89, 1.32) | 1.05 (0.86, 1.28) |
| 80 – 100^th^ percentile | 112 (9.9) | 4,920 (10.0) | 1.02 (0.83, 1.25) | 1.00 (0.81, 1.22) |
| Impaired Water Bodies |  |  |  |  |
| 0 – 19^th^ percentile | 1,021 (90.4) | 42,996 (87.2) | Reference | Reference |
| 20 – 39^th^ percentile | 55 (4.9) | 3,410 (6.9) | 0.68 (0.52, 0.90) | 0.70 (0.53, 0.91) |
| 40 – 59^th^ percentile | 36 (3.20 | 2,011 (4.1) | 0.76 (0.54, 1.06) | 0.73 (0.52, 1.02) |
| 60 – 79^th^ percentile | ^†^ | 434 (0.9) | 0.59 (0.26, 1.31) | 0.55 (0.25, 1.22) |
| 80 – 100^th^ percentile | ^†^ | 432 (0.9) | 1.17 (0.66, 2.06) | 0.96 (0.54, 1.70) |
| Solid Waste |  |  |  |  |
| 0 – 19^th^ percentile | 746 (66.0) | 31,070 (63.0) | Reference | Reference |
| 20 – 39^th^ percentile | 66 (5.8) | 3,482 (7.1) | 0.79 (0.62, 1.02) | 0.73 (0.57, 0.95) |
| 40 – 59^th^ percentile | 75 (6.6) | 3,939 (8.0) | 0.80 (0.63, 1.01) | 0.78 (0.61, 0.99) |
| 60 – 79^th^ percentile | 163 (14.4) | 7,190 (14.6) | 0.95 (0.80, 1.12) | 0.92 (0.78, 1.09) |
| 80 – 100^th^ percentile | 80 (7.1) | 3,602 (7.3) | 0.93 (0.74, 1.17) | 0.90 (0.71, 1.13) |
| **Pollution Burden Score** |  |  |  |  |
| 0 – 19^th^ percentile | ^†^ | 380 (0.8) | Reference | Reference |
| 20 – 39^th^ percentile | 18 (1.6) | 1,025 (2.1) | 1.66 (0.56, 4.90) | 1.91 (0.63, 5.81) |
| 40 – 59^th^ percentile | 83 (7.4) | 4,929 (10.0) | 1.59 (0.58, 4.33) | 1.72 (0.63, 4.71) |
| 60 – 79^th^ percentile | 447 (39.6) | 18,838 (38.2) | 2.23 (0.83, 5.95) | 2.18 (0.81, 5.82) |
| 80 – 100^th^ percentile | 578 (51.2) | 23,989 (48.7) | 2.26 (0.84, 6.04) | 2.26 (0.84, 6.05) |

cRR = crude relative risk

aRR = adjusted relative risk

^*^Adjusted for maternal race/ethnicity, age, education, payment for delivery

^†^ n < 16

NC = not calculated (owing to lack of variability)

## Appendix 3. Crude and adjusted relative risk of early (<34 weeks) preterm birth comparing above versus below the median of environmental exposure by census tract-level socioeconomic status (SES) in Fresno County, 2009-2012 (N=50,413)

|  | **Low SES** | | |  | **High SES** | | | |
| --- | --- | --- | --- | --- | --- | --- | --- | --- |
| **Environmental Exposure** | **< 34 weeks** | **≥37 weeks** |  |  | **< 34 weeks** | **≥37 weeks** |  |  |
|  | **N (%)** | **N (%)** | **cRR (95% CI)** | **aRR**^*^ **(95% CI)** | **N (%)** | **N (%)** | **cRR (95% CI)** | **aRR**^*^ **(95% CI)** |
|  |  |  |  |  |  |  |  |  |
| **Sample** | 612 | 24,998 |  |  | 518 | 24,285 |  |  |
| **Exposures Score** |  |  |  |  |  |  |  |  |
| < 50^th^ | 203 (33.2) | 10,360 (41.4) | Reference | Reference | 285 (55.0) | 14,101 (58.1) | Reference | Reference |
| ≥ 50^th^ | 409 (66.8) | 14,638 (58.6) | 1.41 (1.20, 1.67) | 1.41 (1.19, 1.67) | 233 (45.0) | 10,184 (41.9) | 1.13 (0.95, 1.43) | 1.08 (0.90, 1.28) |
| Ozone |  |  |  |  |  |  |  |  |
| < 50^th^ | 349 (57.0) | 15,063 (60.3) | Reference | Reference | 171 (33.0) | 8,752 (36.0) | Reference | Reference |
| ≥ 50^th^ | 263 (43.0) | 9,935 (39.7) | 1.14 (0.97, 1.34) | 1.14 (0.97, 1.34) | 337 (65.1) | 14,916 (61.4) | 1.15 (0.96, 1.39) | 1.17 (0.97, 1.40) |
| Pesticides |  |  |  |  |  |  |  |  |
| < 50^th^ | 268 (43.8) | 9,872 (39.5) | Reference | Reference | 315 (60.8) | 14,691 (60.5) | Reference | Reference |
| ≥ 50^th^ | 344 (56.2) | 15,126 (60.5) | 0.84 (0.72, 0.99) | 0.85 (0.72, 0.99) | 203 (39.2) | 9,594 (39.5) | 0.99 (0.83, 1.18) | 0.99 (0.83, 1.18) |
| PM_2.5_ |  |  |  |  |  |  |  |  |
| < 50^th^ | 146 (23.9) | 6,434 (25.7) | Reference | Reference | 353 (68.2) | 17,259 (71.1) | Reference | Reference |
| ≥ 50^th^ | 440 (71.9) | 17,286 (69.2) | 1.12 (0.93, 1.34) | 1.11 (0.92, 1.35) | 156 (30.1) | 6,492 (26.7) | 1.17 (0.97, 1.41) | 1.10 (0.91, 1.33) |
| Diesel PM |  |  |  |  |  |  |  |  |
| < 50^th^ | 237 (38.7) | 12,166 (48.7) | Reference | Reference | 229 (44.2) | 12,587 (51.8) | Reference | Reference |
| ≥ 50^th^ | 375 (61.3) | 12,832 (51.3) | 1.49 (1.26, 1.75) | 1.48 (1.26, 1.74) | 289 (55.8) | 11,698 (48.2) | 1.35 (1.13, 1.60) | 1.28 (1.07, 1.52) |
| Toxic Release |  |  |  |  |  |  |  |  |
| < 50^th^ | 154 (25.2) | 8,106 (32.4) | Reference | Reference | 308 (59.5) | 16,391 (67.5) | Reference | Reference |
| ≥ 50^th^ | 458 (74.8) | 16,892 (67.6) | 1.42 (1.18, 1.70) | 1.42 (1.18, 1.70) | 210 (40.5) | 7,894 (32.5) | 1.40 (1.18, 1.67) | 1.31 (1.10, 1.57) |
| Traffic |  |  |  |  |  |  |  |  |
| < 50^th^ | 281 (45.9) | 13,288 (52.9) | Reference | Reference | 220 (42.5) | 11,579 (47.7) | Reference | Reference |
| ≥ 50^th^ | 331 (54.1) | 11,770 (47.1) | 1.32 (1.12, 1.54) | 1.31 (1.12, 1.54) | 298 (57.5) | 12,706 (52.3) | 1.23 (1.03, 1.46) | 1.17 (0.99, 1.39) |
| Drinking Water |  |  |  |  |  |  |  |  |
| < 50^th^ | 33 (5.4) | 1,863 (7.5) | Reference | Reference | 102 (19.7) | 4,713 (19.4) | Reference | Reference |
| ≥ 50^th^ | 579 (94.6) | 23,135 (92.6) | 1.40 (0.99, 1.99) | 1.40 (0.98, 1.98) | 416 (80.3) | 19,572 (80.6) | 0.98 (0.79, 1.22) | 0.96 (0.77, 1.19) |
| *Arsenic* |  |  |  |  |  |  |  |  |
| < 50^th^ | 49 (8.0) | 2,467 (9.9) | Reference | Reference | 129 (24.9) | 6,230 (25.7) | Reference | Reference |
| ≥ 50^th^ | 563 (92.0) | 22,531 (90.1) | 1.25 (0.93, 1.68) | 1.22 (0.91, 1.63) | 389 (75.1) | 18,055 (74.4) | 1.04 (0.85, 1.27) | 1.00 (0.82, 1.22) |
| *Cadmium* |  |  |  |  |  |  |  |  |
| < 50^th^ | 0 (0.0) | 0 (0.0) | Reference | Reference | 0 (0.0) | 0 (0.0) | Reference | Reference |
| ≥ 50^th^ | 612 (100.0) | 24,998 (100.0) | NC | NC | 518 (100.0) | 24,285 (100.0) | NC | NC |
| *1,2-Dibromo-3-chloropropane (DBCP)* | | | |  |  |  |  |  |
| < 50^th^ | 238 (38.9) | 10,100 (40.4) | Reference | Reference | 151 (29.2) | 6,924 (28.5) | Reference | Reference |
| ≥ 50^th^ | 374 (61.1) | 14,898 (59.5) | 1.06 (0.90, 1.25) | 1.06 (0.90, 1.25) | 358 (69.1) | 16,834 (69.3) | 0.98 (0.81, 1.18) | 0.98 (0.81, 1.18) |
| *Hexavalent Chromium* |  |  |  |  |  |  |  |  |
| < 50^th^ | 0 (0.0) | 0 (0.0) | Reference | Reference | 0 (0.0) | 0 (0.0) | Reference | Reference |
| ≥ 50^th^ | 612 (100.0) | 21,281 (85.1) | NC | NC | 518 (100.0) | 24,285 (100.0) | NC | NC |
| *Lead* |  |  |  |  |  |  |  |  |
| < 50^th^ | 0 (0.0) | 0 (0.0) | Reference | Reference | 0 (0.0) | 0 (0.0) | Reference | Reference |
| ≥ 50^th^ | 612 (100.0) | 24,998 (100.0) | NC | NC | 518 (100.0) | 24,285 (100.0) | NC | NC |
| *Nitrate* |  |  |  |  |  |  |  |  |
| < 50^th^ | 243 (39.7) | 10,330 (41.3) | Reference | Reference | 294 (56.8) | 14,410 (59.3) | Reference | Reference |
| ≥ 50^th^ | 369 (60.3) | 14,668 (58.7) | 1.08 (0.91, 1.26) | 1.07 (0.91, 1.26) | 224 (43.2) | 9,875 (40.7) | 1.11 (0.93, 1.32) | 1.08 (0.91, 1.29) |
| *Perchlorate* |  |  |  |  |  |  |  |  |
| < 50^th^ | 0 (0.0) | 0 (0.0) | Reference | Reference | 0 (0.0) | 0 (0.0) | Reference | Reference |
| ≥ 50^th^ | 612 (100.0) | 24,998 (100.0) | NC | NC | 518 (100.0) | 24,285 (100.0) | NC | NC |
| *Trichloroethylene (TCE)* | | |  |  |  |  |  |  |
| < 50^th^ | 237 (38.7) | 11,114 (44.5) | Reference | Reference | 288 (55.6) | 13,607 (56.0) | Reference | Reference |
| ≥ 50^th^ | 375 (61.3) | 13,884 (55.5) | 1.26 (1.07, 1.48) | 1.25 (1.06, 1.47) | 230 (44.4) | 10,678 (44.0) | 1.02 (0.86, 1.21) | 1.00 (0.84, 1.19) |
| *Trichloroethylene (THM)* | | |  |  |  |  |  |  |
| < 50^th^ | 277 (45.3) | 11,838 (47.4) | Reference | Reference | 200 (38.6) | 9,593 (39.5) | Reference | Reference |
| ≥ 50^th^ | 335 (54.7) | 13,160 (52.6) | 1.09 (0.93, 1.27) | 1.06 (0.90, 1.24) | 218 (61.4) | 14,692 (60.5) | 1.04 (0.87, 1.24) | 1.04 (0.87, 1.24) |
| *Uranium* |  |  |  |  |  |  |  |  |
| < 50^th^ | 104 (17.0) | 5,535 (22.1) | Reference | Reference | 70 (13.5) | 3,801 (15.7) | Reference | Reference |
| ≥ 50^th^ | 508 (83.0) | 19,463 (77.9) | 1.38 (1.12, 1.70) | 1.39 (1.13, 1.72) | 424 (81.9) | 18,9488 (78.0) | 1.21 (0.94, 1.56) | 1.19 (0.92, 1.53) |
| *Maximum Contaminant Level (MCL) Violations* | | | |  |  |  |  |  |
| < 50^th^ | 247 (40.4) | 10,577 (42.3) | Reference | Reference | 293 (56.6) | 13,998 (57.6) | Reference | Reference |
| ≥ 50^th^ | 365 (59.6) | 14,4211 (57.7) | 1.08 (0.92, 1.27) | 1.06 (0.90, 1.25) | 225 (43.4) | 10,287 (42.4) | 1.04 (0.88, 1.24) | 1.02 (0.86, 1.21) |
| *Total coliform rule (TCR) Violations* | | | |  |  |  |  |  |
| < 50^th^ | 0 (0.0) | 0 (0.0) | Reference | Reference | 0 (0.0) | 0 (0.0) | Reference | Reference |
| ≥ 50^th^ | 612 (100.0) | 24,998 (100.0) | NC | NC | 518 (100.0) | 24,285 (100.0) | NC | NC |
| **Environmental Effects Score** | | |  |  |  |  |  |  |
| < 50^th^ | 270 (44.1) | 10,073 (40.3) | Reference | Reference | 313 (60.4) | 14,381 (59.2) | Reference | Reference |
| ≥ 50^th^ | 342 (55.9) | 14,925 (59.7) | 0.86 (0.73, 1.01) | 0.85 (0.72, 1.00) | 205 (39.6) | 9,904 (40.8) | 0.95 (0.80, 1.14) | 0.93 (0.78, 1.11) |
| Cleanup Sites |  |  |  |  |  |  |  |  |
| < 50^th^ | 293 (47.9) | 11,805 (47.2) | Reference | Reference | 282 (54.4) | 12,799 (52.7) | Reference | Reference |
| ≥ 50^th^ | 319 (52.1) | 13,193 (52.8) | 0.97 (0.83, 1.14) | 0.96 (0.82, 1.13) | 236 (45.6) | 11,486 (47.3) | 0.93 (0.79, 1.11) | 0.94 (0.79, 1.12) |
| Groundwater Threats |  |  |  |  |  |  |  |  |
| < 50^th^ | 245 (40.0) | 9,545 (38.2) | Reference | Reference | 305 (59.9) | 15,062 (62.0) | Reference | Reference |
| ≥ 50^th^ | 367 (60.0) | 15,453 (61.8) | 0.93 (0.79, 1.09) | 0.91 (0.78, 1.08) | 213 (41.1) | 9,223 (38.) | 1.14 (0.95, 1.35) | 1.12 (0.94, 1.34) |
| Hazardous Waste |  |  |  |  |  |  |  |  |
| < 50^th^ | 248 (40.5) | 10,195 (40.8) | Reference | Reference | 298 (57.5) | 14,430 (59.4) | Reference | Reference |
| ≥ 50^th^ | 364 (59.5) | 14,803 (59.2) | 1.01 (0.86, 1.19) | 1.03 (0.88, 1.21) | 220 (42.5) | 9,855 (40.6) | 1.08 (0.91, 1.28) | 1.02 (0.86, 1.22) |
| Impaired Water Bodies |  |  |  |  |  |  |  |  |
| < 50^th^ | 0 (0.0) | 0 (0.0) | Reference | Reference | 0 (0.0) | 0 (0.0) | Reference | Reference |
| ≥ 50^th^ | 612 (100.0) | 24,998 (100.0) | NC | NC | 518 (100.0) | 24,285 (100.0) | NC | NC |
| Solid Waste |  |  |  |  |  |  |  |  |
| < 50^th^ | 0 (0.0) | 0 (0.0) | Reference | Reference | 0 (0.0) | 0 (0.0) | Reference | Reference |
| ≥ 50^th^ | 612 (100.0) | 24,998 (100.0) | NC | NC | 518 (100.0) | 24,285 (100.0) | NC | NC |
| **Pollution Burden Score** |  |  |  |  |  |  |  |  |
| < 50^th^ | 190 (31.1) | 8,615 (35.5) | Reference | Reference | 346 (66.8) | 16,068 (66.2) | Reference | Reference |
| ≥ 50^th^ | 422 (69.0) | 16,383 (65.5) | 1.16 (0.98, 1.38) | 1.18 (0.99, 1.40) | 172 (33.2) | 8,217 (33.8) | 0.97 (0.81, 1.17) | 0.95 (0.79, 1.15) |

NC = Not Calculated

cRR = crude relative risk

aRR = adjusted relative risk

^*^ Adjusted for maternal race/ethnicity, age, education, payment for delivery

SES defined as “Socioeconomic Factors” score from the CalEnviroScreen, which includes the following variables derived from the US Census American Community Survey: educational attainment, linguistic isolation (households where no one over 14 years of age speaks English very well), poverty and unemployment.

## Appendix 4. Crude and adjusted relative risk of early (<34 weeks) preterm birth comparing above versus below the median of environmental exposure by race/ethnicity in Fresno County, 2009-2012 (N=50,413)

|  | **White non-Hispanic** | | |  | **Non-White*, Non-Hispanic** | | | |
| --- | --- | --- | --- | --- | --- | --- | --- | --- |
| **Environmental Exposure** | **< 34 weeks** | **≥37 weeks** |  |  | **< 34 weeks** | **≥37 weeks** |  |  |
|  | **N (%)** | **N (%)** | **cRR (95% CI)** | **aRR**^†^  **(95% CI)** | **N (%)** | **N (%)** | **cRR (95% CI)** | **aRR**^†^ **(95% CI)** |
|  |  |  |  |  |  |  |  |  |
| **Sample** | 185 | 9,847 |  |  | 311 | 9,840 |  |  |
| **Exposures Score** |  |  |  |  |  |  |  |  |
| < 50^th^ | 102 (55.1) | 5,952 (60.4) | Reference | Reference | 108 (34.7) | 3,676 (37.4) | Reference | Reference |
| ≥ 50^th^ | 83 (44.9) | 3,895 (39.6) | 1.24 (0.93, 1.65) | 1.15 (0.86, 1.54) | 203 (65.3) | 6,164 (62.6) | 1.12 (0.88, 1.41) | 0.99 (0.78, 1.26) |
| Ozone |  |  |  |  |  |  |  |  |
| < 50^th^ | 67 (36.2) | 3,527 (35.8) | Reference | Reference | 126 (40.5) | 4,402 (44.7) | Reference | Reference |
| ≥ 50^th^ | 114 (61.6) | 6,149 (62.5) | 0.98 (0.72, 1.32) | 0.97 (0.82, 1.31) | 185 (59.5) | 5,377 (54.6) | 1.20 (0.95, 1.50) | 1.20 (0.96, 1.50) |
| Pesticides |  |  |  |  |  |  |  |  |
| < 50^th^ | 117 (63.2) | 5,849 (59.4) | Reference | Reference | 201 (64.6) | 6,035 (61.3) | Reference | Reference |
| ≥ 50^th^ | 68 (36.8) | 3,998 (40.6) | 0.85 (0.63, 1.15) | 0.87 (0.65, 1.18) | 110 (35.4) | 3,805 (38.7) | 0.87 (0.69, 1.10) | 0.91 (0.82, 1.15) |
| PM_2.5_ |  |  |  |  |  |  |  |  |
| < 50^th^ | 117 (63.2) | 7,053 (71.6) | Reference | Reference | 151 (48.6) | 4,926 (50.1) | Reference | Reference |
| ≥ 50^th^ | 65 (35.1) | 2,658 (27.0) | 1.46 (1.08, 1.98) | 1.35 (0.99, 1.84) | 157 (50.5) | 4,627 (47.0) | 1.10 (0.88, 1.38) | 1.03 (0.82, 1.29) |
| Diesel PM |  |  |  |  |  |  |  |  |
| < 50^th^ | 85 (44.3) | 5,351 (54.3) | Reference | Reference | 93 (29.9) | 3,891 (39.5) | Reference | Reference |
| ≥ 50^th^ | 103 (55.7) | 4,496 (45.7) | 1.48 (1.11, 1.98) | 1.34 (0.99, 1.80) | 218 (70.1) | 5,949 (60.5) | 1.51 (1.19, 1.93) | 1.33 (1.04, 1.71) |
| Toxic Release |  |  |  |  |  |  |  |  |
| < 50^th^ | 102 (55.1) | 6,961 (70.7) | Reference | Reference | 100 (32.2) | 4,085 (41.5) | Reference | Reference |
| ≥ 50^th^ | 83 (44.9) | 2,886 (29.3) | 1.94 (1.45, 2.59) | 1.78 (1.32, 2.40) | 211 (67.9) | 5,755 (58.5) | 1.48 (1.17, 1.88) | 1.31 (1.02, 1.67) |
| Traffic |  |  |  |  |  |  |  |  |
| < 50^th^ | 85 (46.0) | 5,334 (54.2) | Reference | Reference | 108 (34.7) | 3,960 (40.2) | Reference | Reference |
| ≥ 50^th^ | 100 (54.1) | 4,513 (45.8) | 1.38 (1.04, 1.85) | 1.28 (0.95, 1.71) | 203 (65.3) | 5,880 (59.8) | 1.26 (1.00, 1.59) | 1.14 (0.90, 1.44) |
| Drinking Water |  |  |  |  |  |  |  |  |
| < 50^th^ | 37 (20.0) | 1,953 (19.8) | Reference | Reference | 30 (9.7) | 980 (10.0) | Reference | Reference |
| ≥ 50^th^ | 148 (80.0) | 7,894 (80.2) | 0.99 (0.69, 1.42) | 0.97 (0.68, 1.39) | 218 (90.4) | 8,860 (90.0) | 1.03 (0.71, 1.51) | 0.88 (0.60, 1.29) |
| *Arsenic* |  |  |  |  |  |  |  |  |
| < 50^th^ | 49 (26.5) | 2,843 (28.9) | Reference | Reference | 42 (13.5) | 1,471 (15.0) | Reference | Reference |
| ≥ 50^th^ | 136 (73.5) | 7,004 (71.3) | 1.12 (0.81, 1.56) | 1.10 (0.79, 1.52) | 269 (86.5) | 8,369 (85.1) | 1.12 (0.81, 1.55) | 0.97 (0.70, 1.36) |
| *Cadmium* |  |  |  |  |  |  |  |  |
| < 50^th^ | 0 (0.0) | 0 (0.0) | Reference | Reference | 0 (0.0) | 0 (0.0) | Reference | Reference |
| ≥ 50^th^ | 185 (100.0) | 9,847 (100.0) | NC | NC | 311 (100.0) | 9,840 (100.0) | NC | NC |
| *1,2-Dibromo-3-chloropropane (DBCP)* | | | |  |  |  |  |  |
| < 50^th^ | 53 (28.7) | 3,094 (31.4) | Reference | Reference | 96 (30.9) | 2,891 (29.4) | Reference | Reference |
| ≥ 50^th^ | 29 (69.7) | 6,640 (67.4) | 1.13 (0.82, 1.56) | 1.14 (0.82, 1.56) | 215 (69.1) | 6,909 (70.2) | 0.94 (0.74, 1.19) | 0.89 (0.70, 1.14) |
| *Hexavalent Chromium* |  |  |  |  |  |  |  |  |
| < 50^th^ | 0 (0.0) | 0 (0.0) | Reference | Reference | 0 (0.0) | 0 (0.0) | Reference | Reference |
| ≥ 50^th^ | 185 (100.0) | 9,847 (100.0) | NC | NC | 311 (100.0) | 9,840 (100.0) | NC | NC |
| *Lead* |  |  |  |  |  |  |  |  |
| < 50^th^ | 0 (0.0) | 0 (0.0) | Reference | Reference | 0 (0.0) | 0 (0.0) | Reference | Reference |
| ≥ 50^th^ | 185 (100.0) | 9,847 (100.0) | NC | NC | 311 (100.0) | 9,840 (100.0) | NC | NC |
| *Nitrate* |  |  |  |  |  |  |  |  |
| < 50^th^ | 93 (50.3) | 5,792 (58.8) | Reference | Reference | 133 (42.8) | 4,435 (45.1) | Reference | Reference |
| ≥ 50^th^ | 92 (49.7) | 4,055 (41.2) | 1.40 (1.05, 1.87) | 1.34 (1.00, 1.78) | 178 (57.2) | 5,405 (54.9) | 1.10 (0.87, 1.37) | 0.97 (0.77, 1.22) |
| *Perchlorate* |  |  |  |  |  |  |  |  |
| < 50^th^ | 0 (0.0) | 0 (0.0) | Reference | Reference | 0 (0.0) | 0 (0.0) | Reference | Reference |
| ≥ 50^th^ | 185 (100.0) | 9,847 (100.0) | NC | NC | 311 (100.0) | 9,840 (100.0) | NC | NC |
| *Trichloroethylene (TCE)* | | |  |  |  |  |  |  |
| < 50^th^ | 95 (51.4) | 5,472 (55.6) | Reference | Reference | 116 (37.3) | 3,949 (40.1) | Reference | Reference |
| ≥ 50^th^ | 90 (48.7) | 4,375 (44.4) | 1.18 (0.89, 1.58) | 1.13 (0.84, 1.51) | 195 (62.7) | 5,891 (59.9) | 1.12 (0.89, 1.41) | 0.96 (0.76, 1.22) |
| *Trichloroethylene (THM)* | | |  |  |  |  |  |  |
| < 50^th^ | 63 (34.1) | 3,904 (39.7) | Reference | Reference | 120 (38.6) | 3,591 (36.5) | Reference | Reference |
| ≥ 50^th^ | 122 (66.0) | 5,943 (60.4) | 1.27 (0.93, 1.72) | 1.24 (0.92, 1.68) | 191 (61.4) | 6,249 (63.5) | 0.92 (0.73, 1.15) | 0.86 (0.68, 1.08) |
| *Uranium* |  |  |  |  |  |  |  |  |
| < 50^th^ | 23 (12.4) | 1,781 (18.1) | Reference | Reference | 30 (9.7) | 1,304 (13.3) | Reference | Reference |
| ≥ 50^th^ | 150 (81.1) | 7,293 (74.1) | 1.58 (1.02, 2.45) | 1.60 (1.03, 2.48) | 278 (89.4) | 8,222 (83.6) | 1.45 (1.00, 2.12) | 1.31 (0.89, 1.92) |
| *Maximum Contaminant Level (MCL) Violations* | | | | |  |  |  |  |
| < 50^th^ | 98 (53.0) | 5,704 (57.9) | Reference | Reference | 132 (42.4) | 4,234 (43.0) | Reference | Reference |
| ≥ 50^th^ | 87 (47.0) | 4,143 (42.1) | 1.22 (0.91, 1.63) | 1.17 (0.87, 1.56) | 179 (57.6) | 5,606 (57.0) | 1.02 (0.82, 1.28) | 0.89 (0.70, 1.12) |
| *Total coliform rule (TCR) Violations* | | | |  |  |  |  |  |
| < 50^th^ | 0 (0.0) | 0 (0.0) | Reference | Reference | 0 (0.0) | 0 (0.0) | Reference | Reference |
| ≥ 50^th^ | 185 (100.0) | 9,847 (100.0) | NC | NC | 311 (100.0) | 9,840 (100.0) | NC | NC |
| **Environmental Effects Score** | | |  |  |  |  |  |  |
| < 50^th^ | 112 (60.5) | 5,779 (58.7) | Reference | Reference | 191 (61.4) | 5,668 (57.6) | Reference | Reference |
| ≥ 50^th^ | 73 (39.5) | 4,068 (41.3) | 0.93 (0.69, 1.25) | 0.89 (0.66, 1.20) | 120 (38.6) | 4,172 (42.4) | 0.86 (0.68, 1.08) | 0.86 (0.68, 1.08) |
| Cleanup Sites |  |  |  |  |  |  |  |  |
| < 50^th^ | 101 (54.6) | 5,228 (53.1) | Reference | Reference | 171 (55.0) | 5,144 (52.3) | Reference | Reference |
| ≥ 50^th^ | 84 (45.4) | 4,619 (46.9) | 0.94 (0.71, 1.26) | 0.94 (0.71, 1.26) | 140 (45.0) | 4,696 (47.7) | 0.90 (0.72, 1.13) | 0.91 (0.72, 1.13) |
| Groundwater Threats |  |  |  |  |  |  |  |  |
| < 50^th^ | 100 (54.1) | 5,861 (59.5) | Reference | Reference | 179 (57.6) | 5,456 (55.5) | Reference | Reference |
| ≥ 50^th^ | 85 (46.0) | 3,986 (40.5) | 1.24 (0.93, 1.66) | 1.20 (0.90, 1.61) | 132 (42.4) | 4,384 (44.6) | 0.92 (0.73, 1.15) | 0.92 (0.73, 1.16) |
| Hazardous Waste |  |  |  |  |  |  |  |  |
| < 50^th^ | 98 (53.0) | 5,951 (60.4) | Reference | Reference | 164 (52.7) | 5,170 (52.5) | Reference | Reference |
| ≥ 50^th^ | 87 (47.0) | 3,896 (39.6) | 1.35 (1.01, 1.80) | 1.23 (0.92, 1.66) | 147 (47.3) | 4,670 (47.5) | 0.99 (0.79, 1.24) | 0.96 (0.77, 1.20) |
| Impaired Water Bodies |  |  |  |  |  |  |  |  |
| < 50^th^ | 0 (0.0) | 0 (0.0) | Reference | Reference | 0 (0.0) | 0 (0.0) | Reference | Reference |
| ≥ 50^th^ | 185 (100.0) | 9,847 (100.0) | NC | NC | 311 (100.0) | 9,840 (100.0) | NC | NC |
| Solid Waste |  |  |  |  |  |  |  |  |
| < 50^th^ | 0 (0.0) | 0 (0.0) | Reference | Reference | 0 (0.0) | 0 (0.0) | Reference | Reference |
| ≥ 50^th^ | 185 (100.0) | 9,847 (100.0) | NC | NC | 311 (100.0) | 9,840 (100.0) | NC | NC |
| **Pollution Burden Score** |  |  |  |  |  |  |  |  |
| < 50^th^ | 110 (59.5) | 6,263 (63.6) | Reference | Reference | 158 (50.8) | 4,827 (49.1) | Reference | Reference |
| ≥ 50^th^ | 75 (40.5) | 3,584 (36.4) | 1.19 (0.89, 1.59) | 1.14 (0.85, 1.53) | 153 (49.2) | 5,013 (51.0) | 0.93 (0.75, 1.17) | 0.86 (0.69, 1.08) |

NC = Not Calculated

aRR = adjusted relative risk

* Asian, African-American, Other

^†^ Adjusted for maternal race/ethnicity, age, education, payment for delivery
